# Comparative Performance of Stearoyl Chloride-Modified Cellulose Sponges from Five Lignocellulosic Wastes for Oil/Water Separation

**DOI:** 10.3390/polym18202500

**Published:** 2026-10-13

**Authors:** Emrecan Arpaci, Sami Imamoglu

**Affiliations:** Department of Forest Industry Engineering, Bursa Technical University, 16310 Bursa, Türkiye

**Keywords:** cellulose sponge, lignocellulosic waste, oil adsorption, water treatment, hydrophobic modification

## Abstract

Increasing industrial activity and oil spills have turned oil pollution into a serious threat to aquatic ecosystems. This study compares five lignocellulosic wastes—rice husk, chestnut shell, tissue dust, cotton lint and chemical pulp—as raw materials for chemically modified cellulose sponges as a sustainable oil-removal solution. Cellulose fibres were converted into three-dimensional porous sponges by freezing and lyophilisation, then rendered hydrophobic and oleophilic by esterification with stearoyl chloride, confirmed by a new ester carbonyl band in the FTIR spectra of all five sample series. After modification, static water contact angles increased from a superhydrophilic 0° to 115–136.5°, and the sponges adsorbed oils and organic solvents at 12–17 times their own dry weight, retaining over 70% of this capacity after ten regeneration cycles. Chemical purity (α-cellulose content and lignin/extractive ratio) was the main determinant of performance: sponges from cotton lint and chemical pulp outperformed those from unprocessed agricultural wastes (rice husk, chestnut shell) in hydrophobicity and sorption capacity, while the latter showed compensating gains in mechanical strength. These findings indicate that lignocellulosic-waste-derived cellulose sponges are an environmentally friendly, high-performance sorbent with strong potential for oil-spill clean-up and oily wastewater treatment, with raw material choice guided by the intended application’s priorities.

## 1. Introduction

Various industrial processes, such as food processing, metallurgy, petrochemicals and oil refining, generate significant volumes of oily wastewater every day. The impact of these wastewaters on aquatic ecosystems is long-lasting and destructive, as is clearly demonstrated by the consequences of frequently occurring oil-spill accidents [1]. The rapidly growing world population and worsening climate conditions have turned freshwater scarcity into a global problem, a situation that has become especially pronounced in water-scarce regions [2,3,4]. These environmental concerns make the effective treatment of oil/water mixtures imperative; compared with disposal methods such as incineration, oil recovered through artificial separation can be collected and reused without causing additional pollution [5].

Although conventional techniques such as gravity separation, skimming and flotation have been widely used for oil/water separation, they suffer from significant drawbacks, including high cost, the use of toxic components, the formation of secondary pollutants, and the need for large installation areas. These limitations have brought membrane separation processes to the fore as an emerging technology [6]. Membrane-based methods are highly attractive owing to the wide variety of materials available that offer high energy efficiency and selective filtration [7]; accordingly, materials such as PDMS-coated nanowire membranes, PTFE-coated metal meshes and cross-linked polymer gels have been developed [8,9,10]. However, these “oil-removing” materials tend to foul and clog easily owing to their oleophilic nature [11], and most require external pumping systems, which increases energy consumption. These drawbacks have shifted research attention towards hydrophilic/oleophobic filters [12]; however, most existing hydrophilic/oleophobic filters are made from non-renewable synthetic materials that are difficult to dispose of [13].

At this point, cellulose and its derivatives stand out as an environmentally friendly alternative owing to “green” properties such as availability, renewability, biocompatibility and biodegradability [14]. Nanocellulose and cellulose-based porous structures (sponges, aerogels) are attracting increasing interest in water-treatment applications owing to their high surface area and low density [15]. Functionalising cellulose through carboxymethylation, sulfonation or long-chain acyl groups alters the hydrophilic nature of the material, rendering it suitable for pollutant adsorption [16]. However, a substantial proportion of previously developed cellulosic membranes have been modified with harmful fluorine-containing chemicals to impart oleophobic behaviour [17], which still calls for improvement in order to reduce environmental impact.

On the other hand, oily wastewater constitutes a significant proportion of industrial discharges and contains oil in free, dispersed, emulsified and dissolved forms [18,19]. The petrochemical and metalworking industries are among the main sources of these wastewaters; the volume of oily wastewater generated worldwide is estimated to reach 9–14 billion m^3^ [20]. Water pollution poses a serious threat to public health through unsafe water sources and leads to toxic pollutants accumulating in the food chain and ultimately reaching human health [21,22]. Mineral oils, which are commonly used in metalworking wastewaters, are also a significant contributor to this pollution burden [23].

This research aims to minimise the environmental impact of chemically modified cellulosic sponges intended specifically for the removal of oil pollution from water. Within this scope, the objectives of the study can be listed as follows: (i) obtaining high-value-added cellulose from various lignocellulosic wastes; (ii) determining the physical, chemical and mechanical properties of the raw materials used and the sponges produced; (iii) converting the resulting cellulose fibres into a three-dimensional, porous sponge structure; (iv) rendering the sponges hydrophobic and oleophilic through chemical modification with stearoyl chloride; and (v) measuring the efficiency of the modified sponges in separating oil/water mixtures and their performance in adsorbing various chemicals.

## 2. Materials and Methods

### Raw Materials and Chemical Analyses

**Figure 1:**
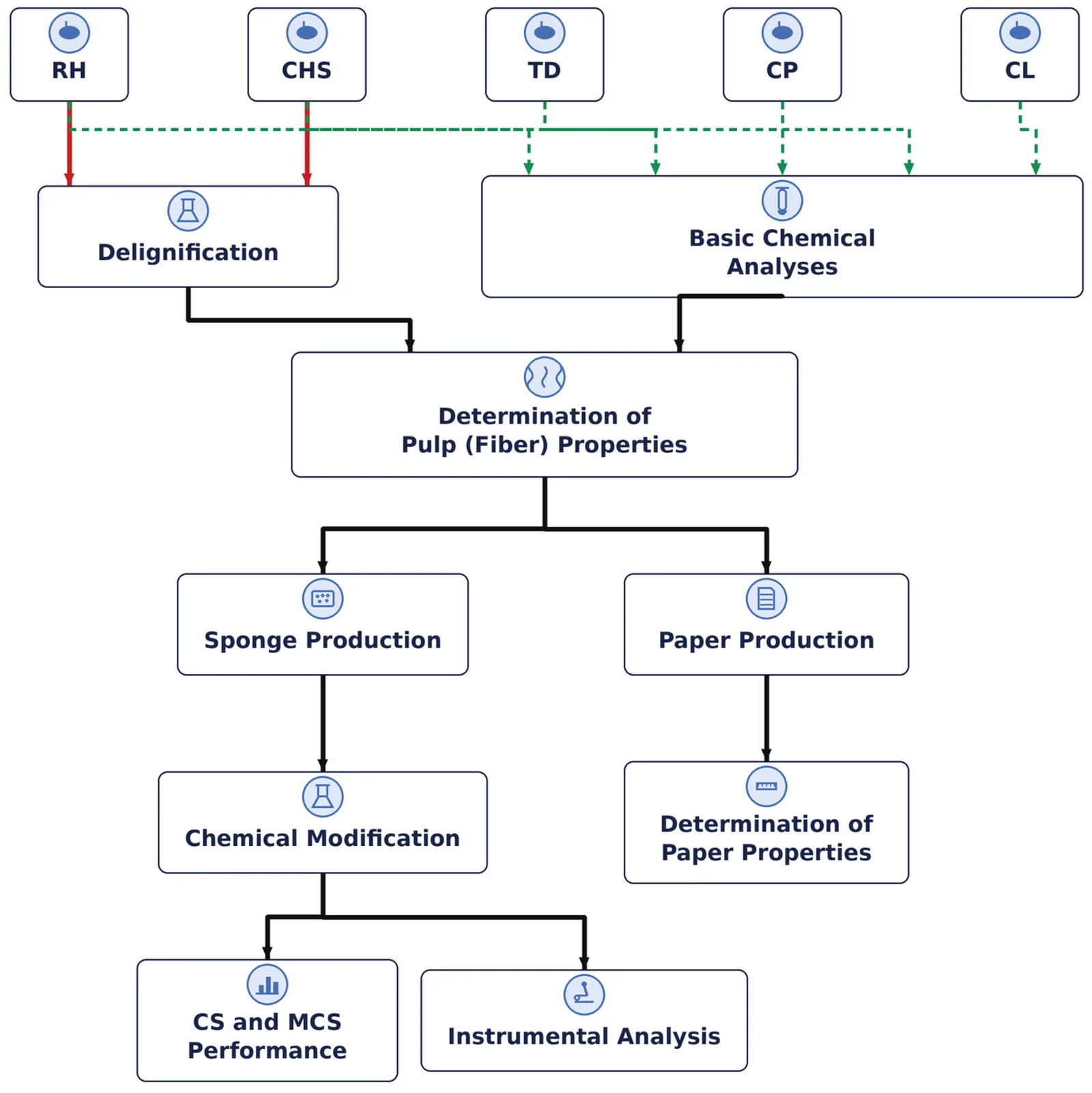
Overall workflow of the study: from raw material sourcing and chemical analyses, through cellulose production, sponge production/modification, and instrumental characterisation.

**Figure 2:**
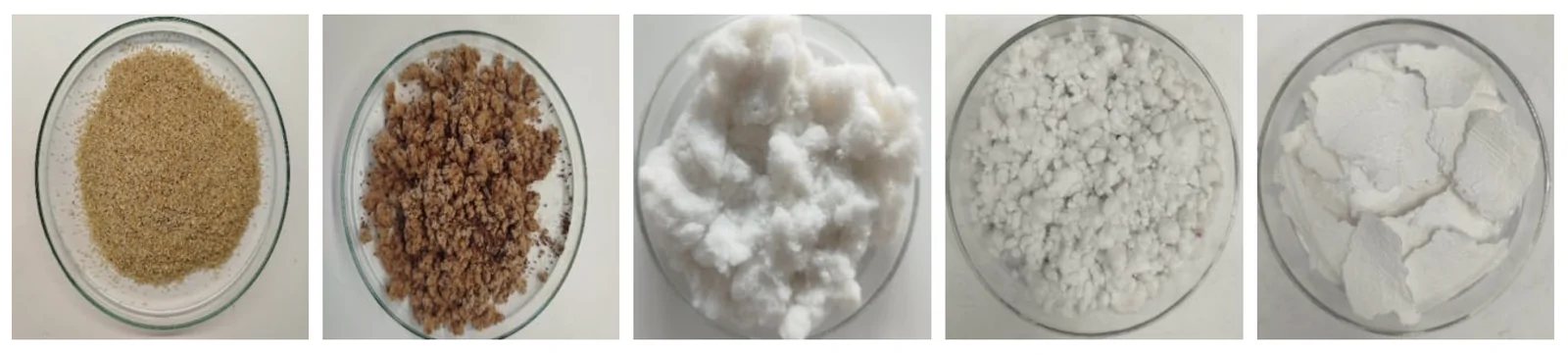
Ground raw materials used in the study (**left** to **right**): RH, CHS, CL, TD and CP.

Five different lignocellulosic sources were used in this study for the production of cellulose-based sponges: rice husk (RH), chestnut shell (CHS), tissue dust (TD), cotton lint (CL) and chemical pulp (CP). Rice husks were obtained from a rice mill, chestnut shells from a candied-chestnut factory, tissue dust and chemical pulp from a tissue-paper mill, and cotton lint from a yarn manufacturer. The samples were ground and sieved to a 40–100 mesh range to prepare them for analysis and delignification. The overall workflow of the study is presented in Figure 1, while the ground forms of the five raw materials used are shown in Figure 2.

The cellulose, hemicellulose, lignin, inorganic matter (ash) and extractive contents of the raw materials were determined using standard lignocellulosic biomass analysis methods (TAPPI T 208 om-94, T 211 om-93, T 212 om-98, T 207 om-88, T 204 om-88, T 222 om-02). Klason and acid-soluble lignin values were summed to obtain total lignin, while holocellulose and α-cellulose contents were calculated using the Wise method and 17.5% NaOH treatment, respectively. As tissue dust, cotton lint and chemical pulp consist almost entirely of cellulose, they were not subjected to delignification; rice husk and chestnut shell samples, however, were subjected to a multi-stage delignification process using 5% NaOH, 10% HCl, 2% NaClO2 (pH 4) and 5% Na_2_SO_3_ treatments, respectively, to obtain value-added cellulose.

**Figure 3:**
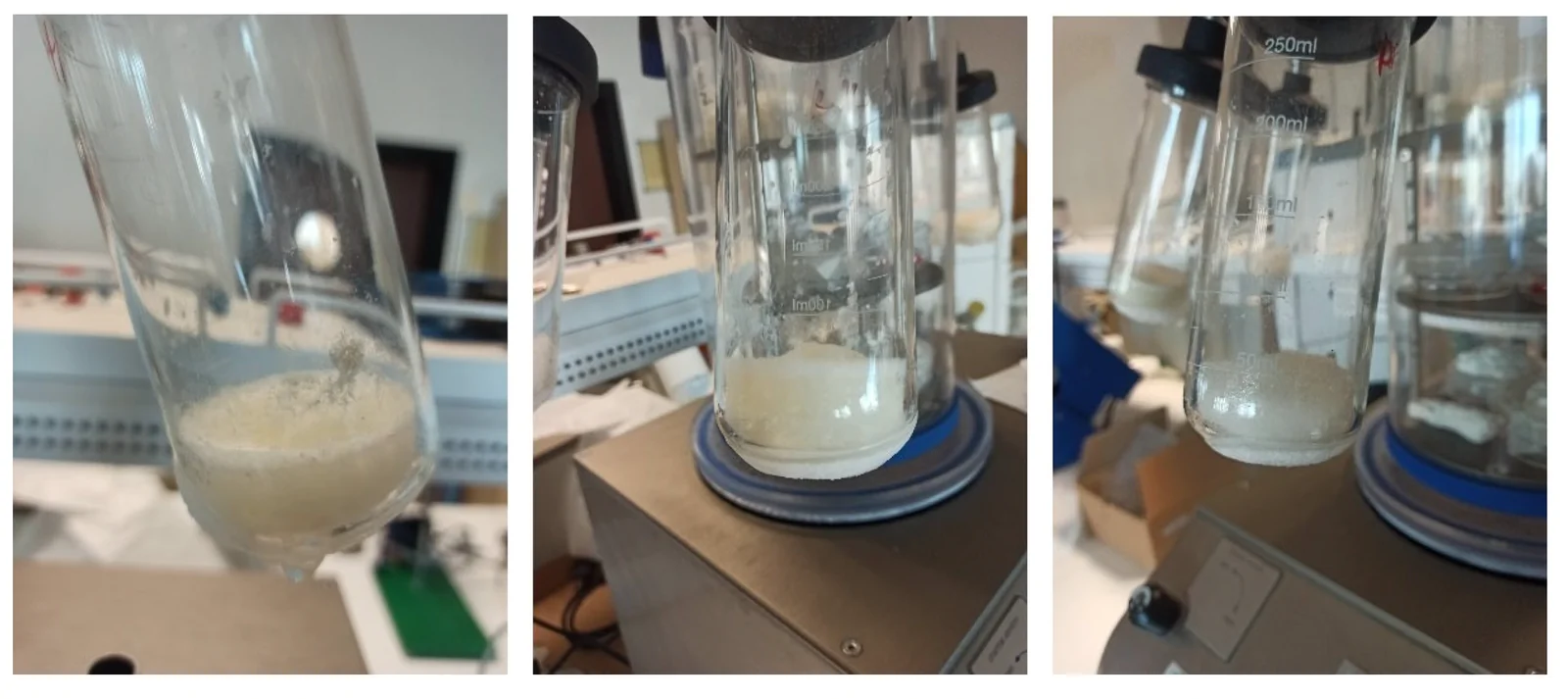
Forming cellulose pulps into sponges: views of the cellulose suspension in centrifuge tubes at successive stages of loading into the deep-freeze cabinet prior to lyophilisation.

**Figure 4:**
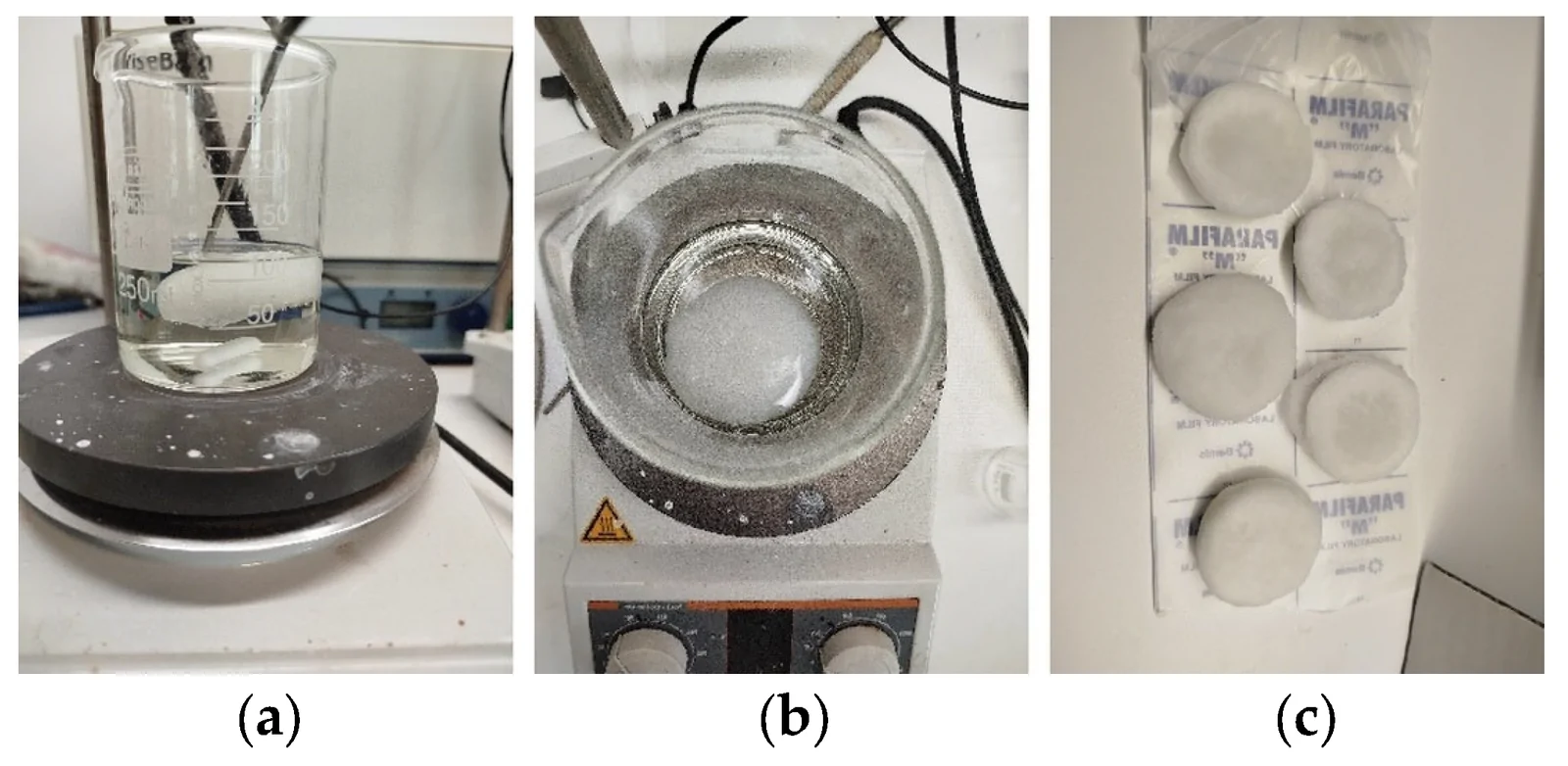
CS modification process and MCS samples after washing: (**a**) CS immersed in the stearoyl chloride reaction solution on a magnetic stirrer; (**b**) the reaction vessel during esterification at 45 °C; (**c**) the resulting circular MCS samples after hexane washing and drying, individually packaged for storage.

The resulting cellulose fibres were converted into three-dimensional, porous sponge structures (CS) by freezing the aqueous suspensions, prepared at 2% consistency, at −40 °C and drying them in a lyophiliser (freeze-drying/lyophilisation) (Figure 3). To render the naturally hydrophilic surface of the sponges hydrophobic and oleophilic, the sponges were subjected to an esterification reaction in a 0.2 M stearoyl chloride solution at 45 °C for 1 h; the modified sponges (MCS) were then washed with hexane and dried in a vacuum oven at 50 °C (Figure 4).

The performance of the produced CS and MCS samples was evaluated through static contact-angle measurements, water/olive oil/ethanol/toluene/synthetic motor oil adsorption capacity tests, and centrifugation-based reusability trials (10 cycles, 4000 rpm, 240 s). For contact-angle measurements, sponge samples were cut to a uniform 10 × 10 mm cross-section; sample height was not fixed and varied naturally between approximately 5 and 10 mm across the different raw-material series, since the pore architecture produced by lyophilisation, and hence the resulting sponge height at a given suspension volume, differs inherently with fibre morphology. In addition, test papers with a grammage of 65 g/m^2^ were produced from the obtained fibres using a Rapid Köthen handsheet former (HAAGE, Peissenberg, Germany), and their freeness, pH, thickness/density, tensile and burst strength, colour, surface roughness and air permeability were determined. The chemical structure and morphological properties of the materials were characterised by FTIR (Bruker Tensor37 (Bruker, Billerica, MA, USA), 400–4000 cm^−1^), simultaneous TGA/DTA (Hitachi STA7200 (Hitachi, Tokyo, Japan), 10–800 °C, 10 °C/min, N_2_ atmosphere), SEM (Carl Zeiss Gemini 301 (Carl Zeiss, Oberkochen, Germany), Au/Pd coating) and XRD (Bruker D8 Discovery, Cu Kα, 2θ = 10–90°, evaluated over the diagnostic 10–40° range) analyses. To verify accuracy, each test was performed in at least duplicate for every sample, and any test that yielded an inconsistent result was repeated to rule out possible human or instrumental error; the values reported in this study represent these repeated determinations.

## 3. Results and Discussion

### 3.1. Chemical Composition of the Raw Materials

The basic chemical composition of the five different lignocellulosic raw materials used in the study (RH, CHS, TD, CL, CP) was determined by measuring moisture, ash, 1% NaOH solubility, hot-water solubility, total lignin, holocellulose and α-cellulose content, and is summarised in Table 1.

In terms of moisture content, the samples fell within a similarly low range (6.5–9.3%), with the highest value recorded for RH (9.3%) and the lowest for CP (6.5%); this difference was attributed to the storage conditions and natural hygroscopicity of the raw materials. Ash content, on the other hand, varied over a much wider range (0.8–16.2%) depending on the raw material source. RH, of agricultural-waste origin, exhibited the highest ash content (16.2%) owing to its high biogenic silica (SiO_2_) accumulation [24], whereas industrially purified CP was almost entirely free of inorganic matter, yielding the lowest ash value (0.8%). TD (11.5%) and CL (5.8%) showed intermediate values, while CHS (2.1%) stood out with a low ash content close to that of CP.

When 1% NaOH solubility is considered as an indicator of hemicellulose and degraded low-molecular-weight carbohydrates, the highest solubility was observed in CHS (18.0%) and TD (15.5%), indicating that both sources contain substantial amounts of hemicellulose and easily hydrolysable components. CP (6.2%) and RH (7.9%) showed the lowest NaOH solubility values, indicating a more stable carbohydrate structure. Hot-water solubility results were strikingly high for CHS (26.0%), confirming that chestnut shell is rich in tannins and other water-soluble phenolic extractives. All other raw materials exhibited hot-water solubility below 5% (RH 0.30%; TD 4.5%; CL 3.1%; CP 1.8%), indicating a relatively poor structure in terms of such water-soluble components.

Total lignin (the sum of Klason and acid-soluble lignin) and holocellulose/α-cellulose results provided the most decisive information regarding the cellulosic purity of the raw materials. The highest total lignin content was detected in CHS (33.5%), showing that chestnut shell contains not only extractives but also a dense lignin network. RH likewise exhibited a relatively high lignin ratio (21.30%). In contrast, TD (3.80%), CL (2.50%) and CP (0.80%) were largely free of lignin owing to having undergone industrial-type chemical pulping/bleaching processes, a finding that directly corresponded with their high holocellulose (95.00%, 95.80% and 98.50%, respectively) and α-cellulose (86.50%, 93.50% and 90.20%, respectively) values. The lower holocellulose (55.81% and 35.00%) and α-cellulose (40.47% and 21.00%) ratios of RH and CHS confirmed that these two agricultural wastes contain substantial amounts of lignin, non-hemicellulose components and inorganic matter prior to delignification, and therefore require a multi-stage delignification process to obtain value-added cellulose. Overall, industrially purified chemical pulp (CP) offered the purest cellulosic structure, with the lowest ash and the highest holocellulose/α-cellulose content, while the agricultural wastes (RH, CHS) displayed a more heterogeneous profile with high hemicellulose, lignin and inorganic-matter contents. These findings form the chemical basis for the performance differences discussed in Section 3.2, Section 3.3, Section 3.4, Section 3.5 and Section 3.6.

The extractive content of the raw materials was determined using a two-stage extraction procedure (alcohol-cyclohexane and ethanol), and the total values have been added to Table 1. The highest total extractive content, 22.00%, was found in CHS, confirming the richness of chestnut shell in tannin-like polyphenolic compounds [25]. The fact that most of RH’s total extractive content (3.45%) dissolved in the non-polar alcohol-cyclohexane phase indicated that the husk is rich in natural oils, waxes and lipophilic compounds. CL (1.50%), TD (1.00%) and, in particular, CP (0.50%) showed the lowest extractive contents owing to the purification steps they underwent during industrial processing.

### 3.2. Surface Wettability and Contact Angle

**Figure 5:**
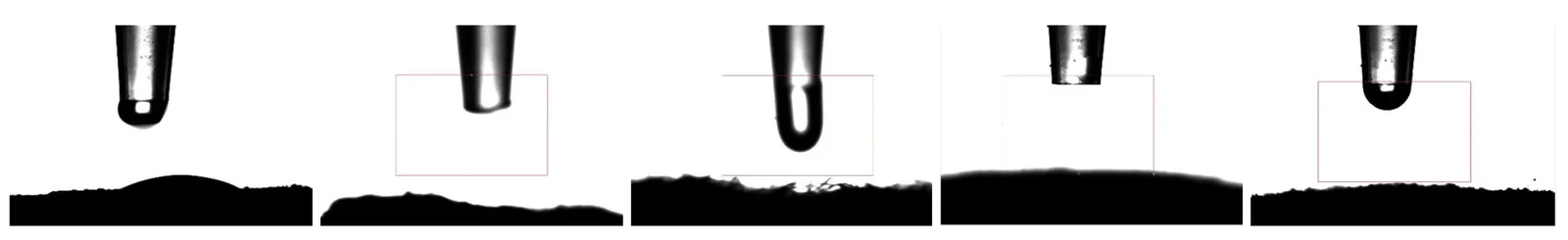
Images showing that water droplets placed on CS samples were instantly absorbed, leaving no measurable contact angle (**left** to **right**: CS-RH, CS-CHS, CS-CL, CS-TD, CS-CP).

**Figure 6:**
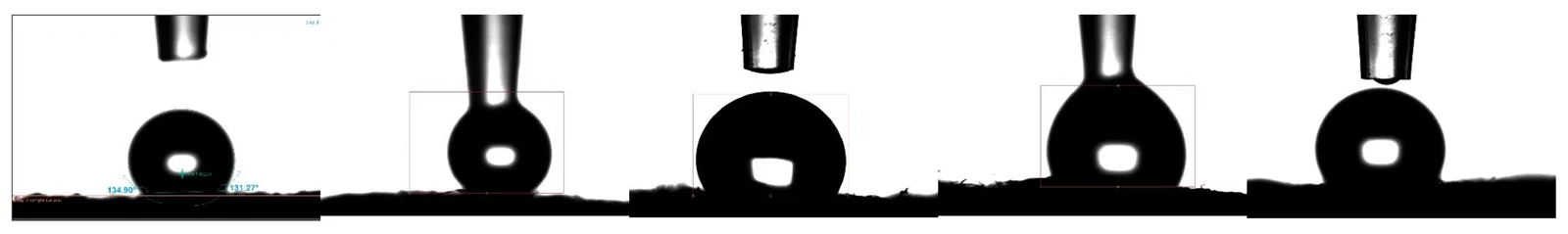
Static contact-angle images of MCS samples (**left** to **right**: MCS-CL, MCS-TD, MCS-RH, MCS-CHS, MCS-CP).

In contact-angle trials performed on unmodified cellulose sponge (CS) samples, the water droplet was absorbed instantly upon contact with the surface and no measurable angle could be obtained (θ ≈ 0°; Figure 5); this is direct evidence of the superhydrophilic character exhibited by cellulose owing to its abundant polar -OH groups. Following esterification with stearoyl chloride, however, a radical change in surface properties was observed, with all MCS samples exhibiting high static contact-angle values in the range of 115–136.5° (Figure 6, Table 2; Section 3.5). The highest hydrophobicity was obtained for the MCS-CL sample (136.5°), produced from long, pure cotton fibres, while the lowest value was measured for the MCS-CP sample (115.0°). This transformation is explained as a result of the esterification reaction in which the hydroxyl groups on the cellulose surface are replaced by long, non-polar C18 stearoyl chains.

**Figure 7:**
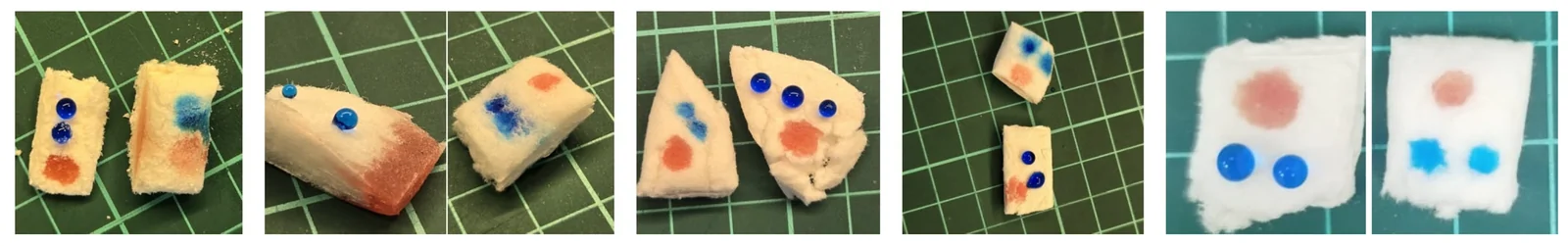
Comparative images of water (blue) and oil (red) droplets placed on CS and MCS sponges—**left** to **right**: samples derived from RH, CHS, TD, CP and CL.

The qualitative visual droplet analysis, performed using methylene-blue-dyed water and oil-red-dyed olive oil droplets, strikingly visualised the surface transformation on samples produced from five different raw material sources (RH, CHS, TD, CP, CL) (Figure 7). A common pattern was observed across all samples: blue water droplets placed on the unmodified sponge surface were completely absorbed and spread across the surface within seconds, while red oil droplets placed on the same surface likewise showed limited, superficial spreading. After modification, however, blue water droplets remained on the surface, retaining their spherical shape, while red oil droplets were absorbed rapidly and homogeneously throughout the pores. The fact that this behaviour was consistently observed across all RH, CHS, TD, CP and CL samples, irrespective of raw material source, visually demonstrates that the selective (hydrophobic/oleophilic) wetting character imparted to the sponge by the esterification reaction is not specific to a single sample, and that the developed modification procedure is generalisable to different lignocellulosic sources.

### 3.3. Water, Oil and Organic Solvent Adsorption Capacity

The water absorption capacities, determined using a procedure adapted from the standard Cobb test (ISO 535) [26], are among the results that most strikingly demonstrate the effectiveness of the modification (Table 3). Unmodified sponges (CS) absorbed 12–15 times their own weight in water (1180–1450%) owing to the natural hydrophilicity of cellulose and the high porosity created by lyophilisation. Following stearoyl chloride modification, these values dropped dramatically to the 26–54% range. The lowest water absorption values (indicating the most successful hydrophobic transformation) were again observed for MCS-CL (26%) and MCS-CP (31%), while the highest residual water absorption ratios were measured for the impurity-containing MCS-RH (48%) and MCS-CHS (35%).

It is worth noting that the relative decrease in water absorption upon modification was of a broadly similar order across all five series, even though the raw materials differed substantially in purity and fibre morphology; water repellency therefore appears to plateau rapidly once the stearoyl chloride grafting reaction succeeds, rather than scaling proportionally with feedstock purity. Consequently, the water absorption ratio alone is not a sufficient criterion for selecting the best-performing raw material. As shown in the following sections, the more discriminating criteria are the oil/organic-solvent adsorption capacity, the mechanical durability of the derived paper samples, and the retention of adsorption capacity over repeated regeneration cycles, all of which varied far more markedly between feedstocks than the water absorption ratio and therefore provide the practical basis for raw-material selection in a given application.

**Figure 8:**
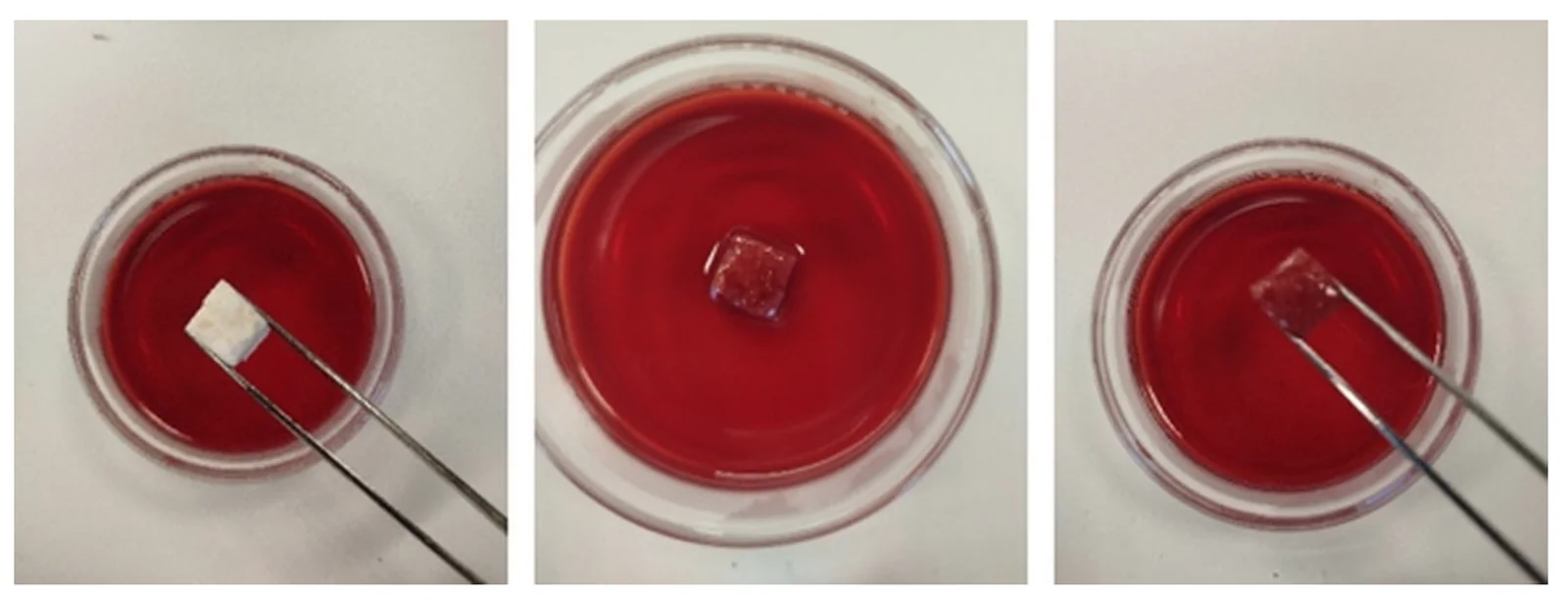
Photographs from the adsorption test performed with red-dyed olive oil: immersion of the sponge sample in oil and the process of oil adsorption by the sponge.

Adsorption experiments carried out with olive oil, ethanol, toluene and synthetic motor oil quantitatively demonstrated the selectivity imparted by the chemical modification towards non-polar liquids (Table 4, Table 5, Table 6 and Table 7; Figure 8; see Section 3.6.4. for a consolidated comparison across all four liquids). Unmodified sponges (CS) exhibited relatively low non-polar liquid adsorption owing to their hydrophilic surface character; this limited uptake largely resulted from liquid physically filling the macropores of the material. MCS samples, in contrast, exhibited an extraordinary absorption capacity corresponding to 12 to 17 times their own dry weight. The highest performance was obtained for the MCS-CL and MCS-CP samples, which possess the purest and most hydrophobic surfaces; this is explained by the strong van der Waals interactions established between the stearoyl chains grafted onto the surface and non-polar oil/solvent molecules [27], together with the entrapment of liquid by the high-volume pore network created through lyophilisation. The sponges’ high affinity for the amphiphilic liquid ethanol (1010–1180% for CS, 1110–1420% for MCS) was associated with the capillary effect of the material’s high porosity; this finding indicates that the developed material can be used as a versatile sorbent not only for oils but also for various organic solvents. In tests with synthetic motor oil, an industrially significant pollutant, MCS samples likewise exhibited high capacity in the 1240–1580% range, confirming that the material has a broad range of effectiveness against petroleum derivatives of differing viscosity and chemical structure.

Comparing these values against the literature helps contextualise the performance achieved here. Phanthong et al. [28] reported that a nanocellulose sponge, hydrophobised with the same stearoyl chloride agent under near-identical conditions (50 °C, 1 h), achieved absorption capacities of 25–55 times its own dry weight and 160° water contact angles, both moderately higher than the 12–17 times and 115–136.5° obtained in the present study. Shang et al. [29], using a different hydrophobisation route (chemical vapour deposition with hexadecyltrimethoxysilane) on a cross-linked nanocellulose aerogel, reported considerably higher absorption capacities of 77–226 g/g together with strong retention (170 g/g for chloroform) after 30 cycles. Taken together, these comparisons suggest that the sponges developed in this study, while markedly less absorbent than nanocellulose-based aerogels prepared from purified, nanoscale feedstocks, achieve a comparable order of hydrophobic transformation and a similar or better reusability profile (10 cycles at 70.4–87.5% retention) using coarser, waste-derived lignocellulosic fibres and a simpler, single-step esterification without any nanofibrillation step. This trade-off between absolute sorption capacity and raw material simplicity/cost should be weighed according to the intended application.

### 3.4. Reusability

**Figure 9:**
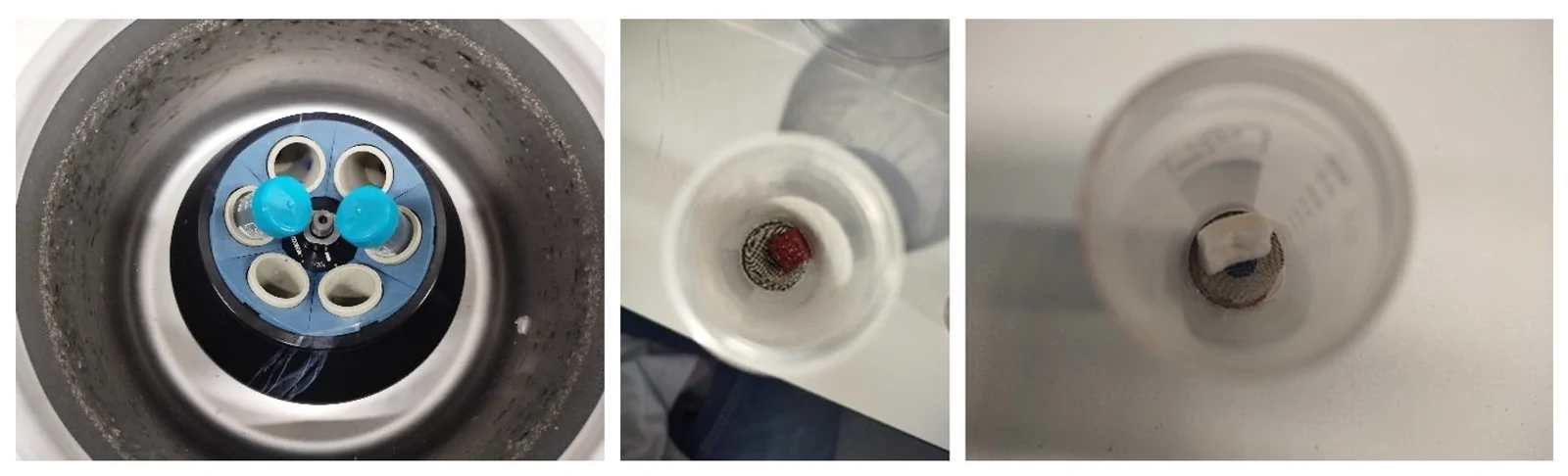
The centrifuge apparatus used in the reusability tests, and oil-saturated sponge samples placed in centrifuge tubes (before and after centrifugation).

The economic and ecological sustainability of the developed sorbent materials in practical applications depends largely on their regeneration capability. Accordingly, after 10 consecutive adsorption-regeneration cycles performed through mechanical squeezing and low-speed centrifugation (4000 rpm, 240 s; Figure 9), the cotton-derived MCS-CL sample showed the highest stability, retaining 87.5% of its initial capacity (Table 8; Section 3.6.4). MCS-CP (84.5%) and MCS-CHS (81.5%) samples likewise exhibited high cyclic stability, whereas capacity loss was more pronounced in the MCS-TD (74.7%) and MCS-RH (70.4%) samples, which have a high fines content. These results show that sponges produced from long, processed fibres (CL, CP, CHS) form a three-dimensional network structure that is more resistant to repeated mechanical stress, and that raw material selection is a critical parameter for reusability.

### 3.5. Physical and Mechanical Properties of the Paper Samples

**Figure 10:**
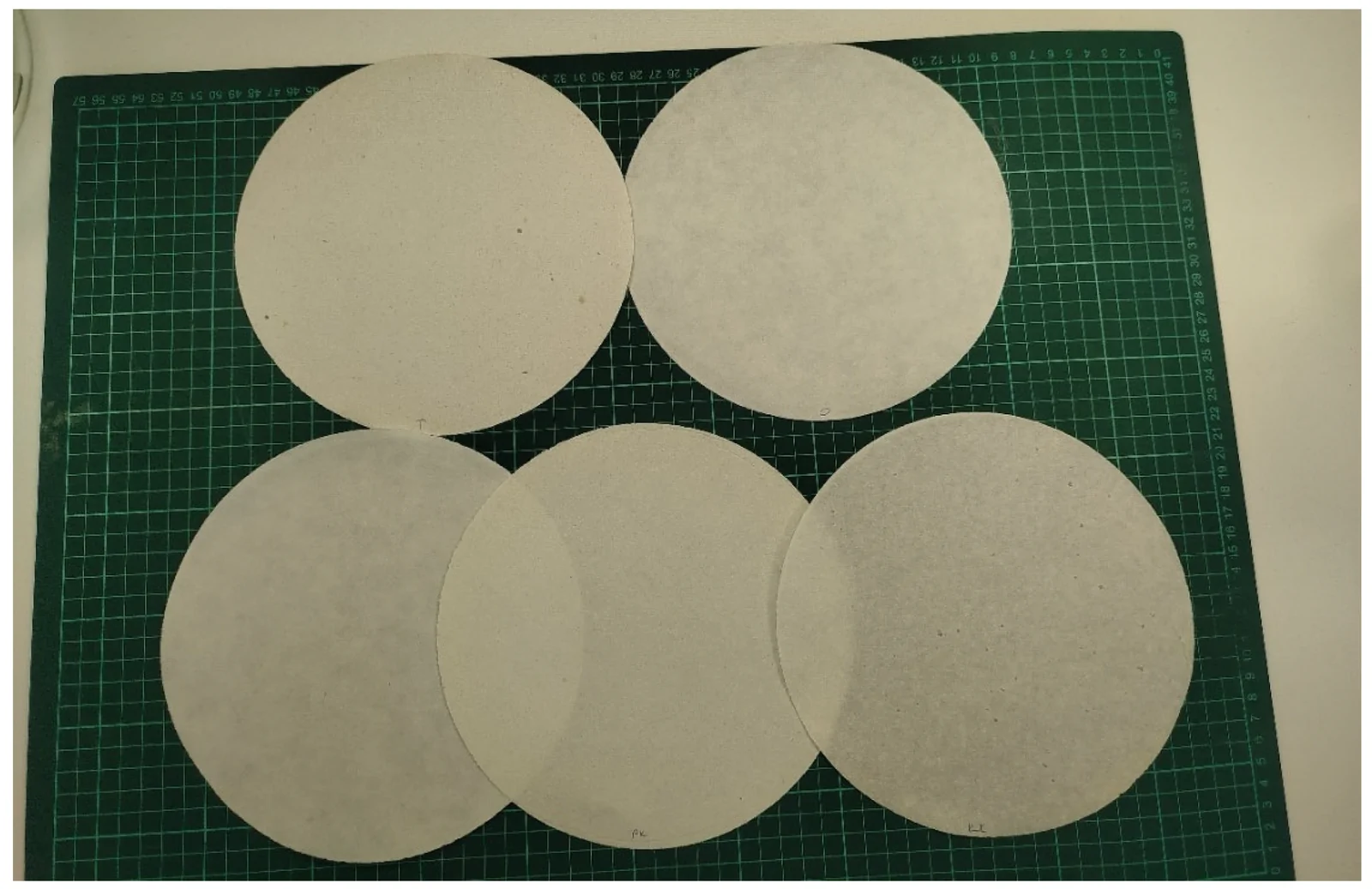
Handsheet test papers (65 g/m^2^) produced from different raw materials using the Rapid Köthen method.

**Figure 11:**
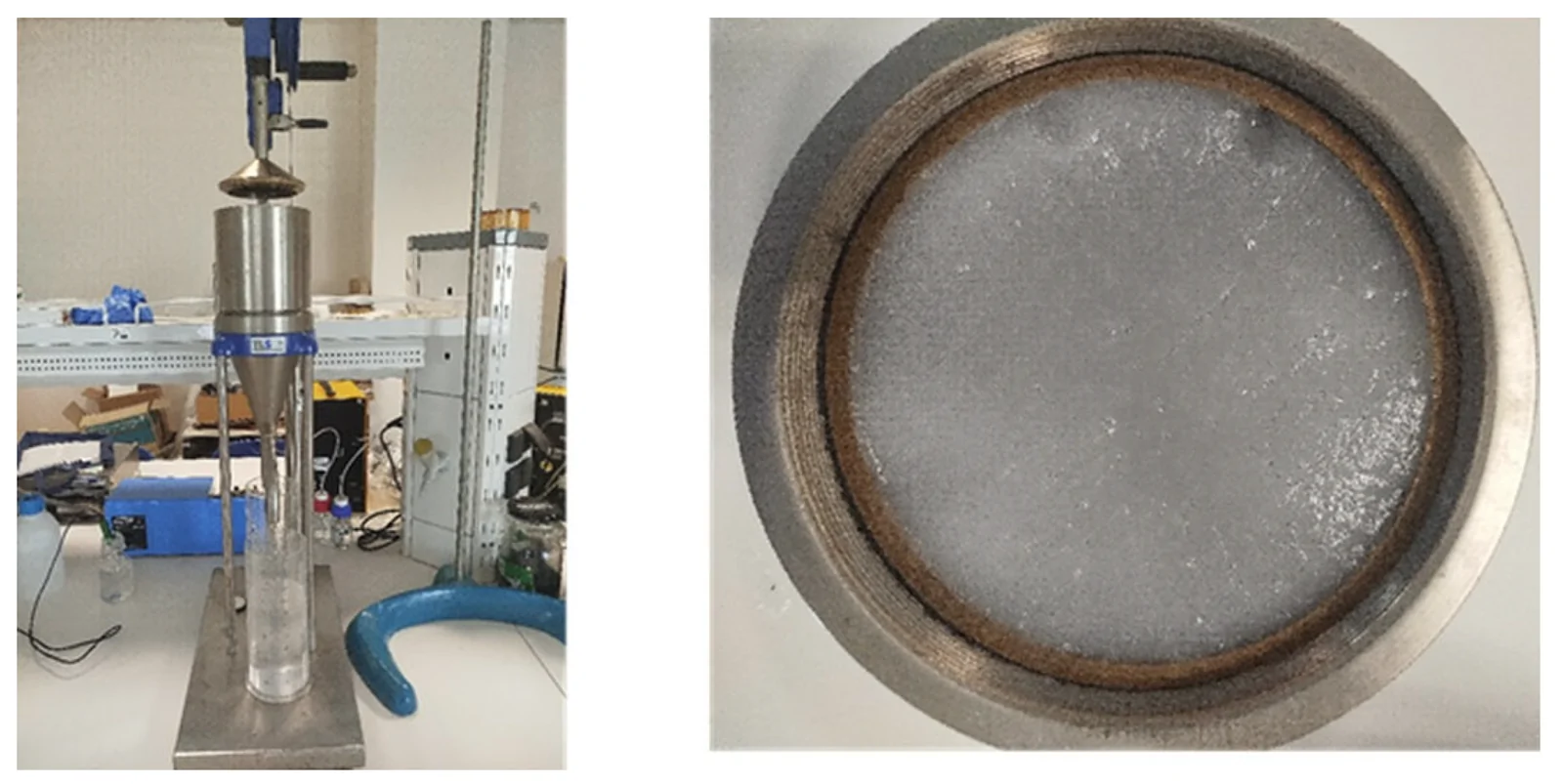
Schopper-Riegler freeness measurement apparatus (TLS, Techlab Systems, Lezo, Spain).

In order to indirectly simulate the mechanical strength of the sponges, handsheet test papers were produced under standard conditions (65 g/m^2^) from the fibres obtained from each raw material (Figure 10), and the freeness, pH, structural and mechanical properties of these papers were comparatively examined (Table 9, Table 10, Table 11, Table 12, Table 13 and Table 14, Figure 11).

**Figure 12:**
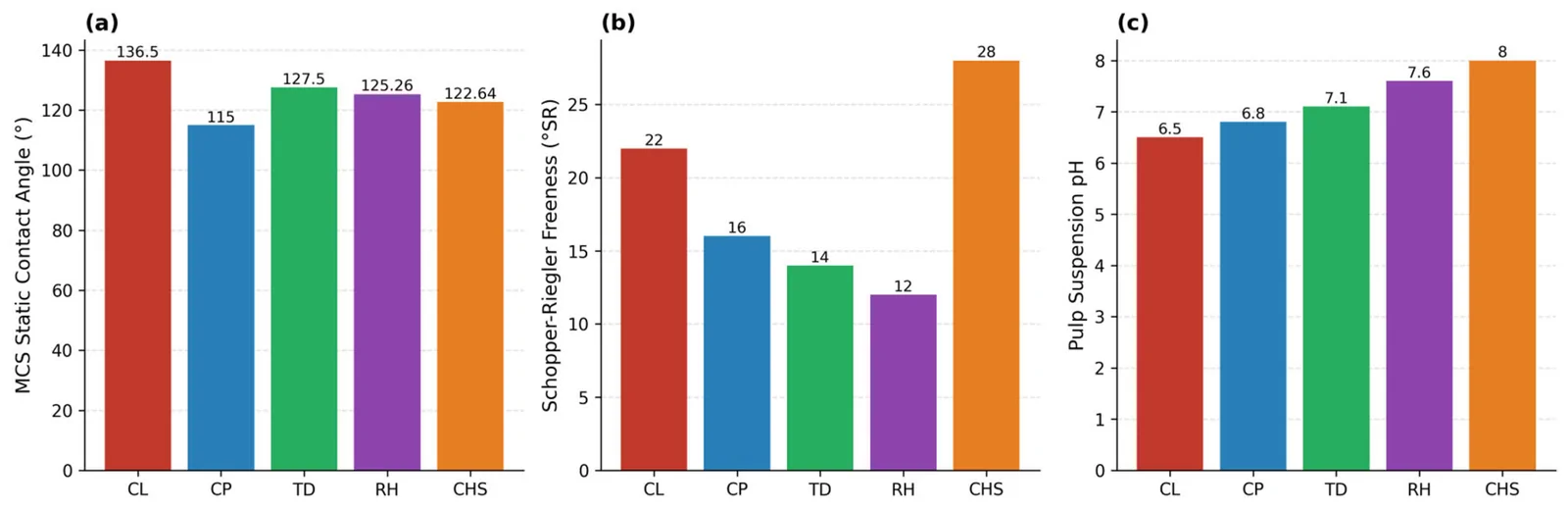
Comparative summary of pulp processing properties across all five raw-material series: (**a**) MCS static contact angle (consolidated from Table 2); (**b**) Schopper-Riegler freeness of the cellulosic pulps (consolidated from Table 9); (**c**) pulp suspension pH (consolidated from Table 10).

The lowest °SR values were observed for RH (12) and TD (14) (Figure 12b); this indicates that both sources consist of short fibres with low fibrillation potential. In contrast, CL (22) and, in particular, CHS (28) exhibited freeness values above what would be expected for unbeaten pulps, owing to their high hemicellulose and fines content.

The pH values of all pulp suspensions were measured within a range close to neutral (6.5–8.0). CHS (8.0) and RH (7.6) were the most alkaline pulps (Figure 12c), while CL (6.5) exhibited a slightly acidic profile; these differences were attributed to the natural extractive content of the raw materials and the chemical treatments applied.

The highest bulk (2.16 cm^3^/g) was observed in the CL sample, which is composed of long, flexible cotton fibres; this points to a porous, low-density network structure. RH (1.51 cm^3^/g) and CHS (1.43 cm^3^/g), of agricultural-waste origin, exhibited a more compact, dense structure owing to their high fines content.

The highest breaking length was measured for CHS (4.8 km) and RH (4.5 km), but these samples exhibited brittle behaviour with only 1.6–1.8% elongation. CL, in contrast, displayed a more ductile mechanical profile with the highest elongation (3.5%); this confirms the well-known trade-off between strength and flexibility.

**Figure 13:**
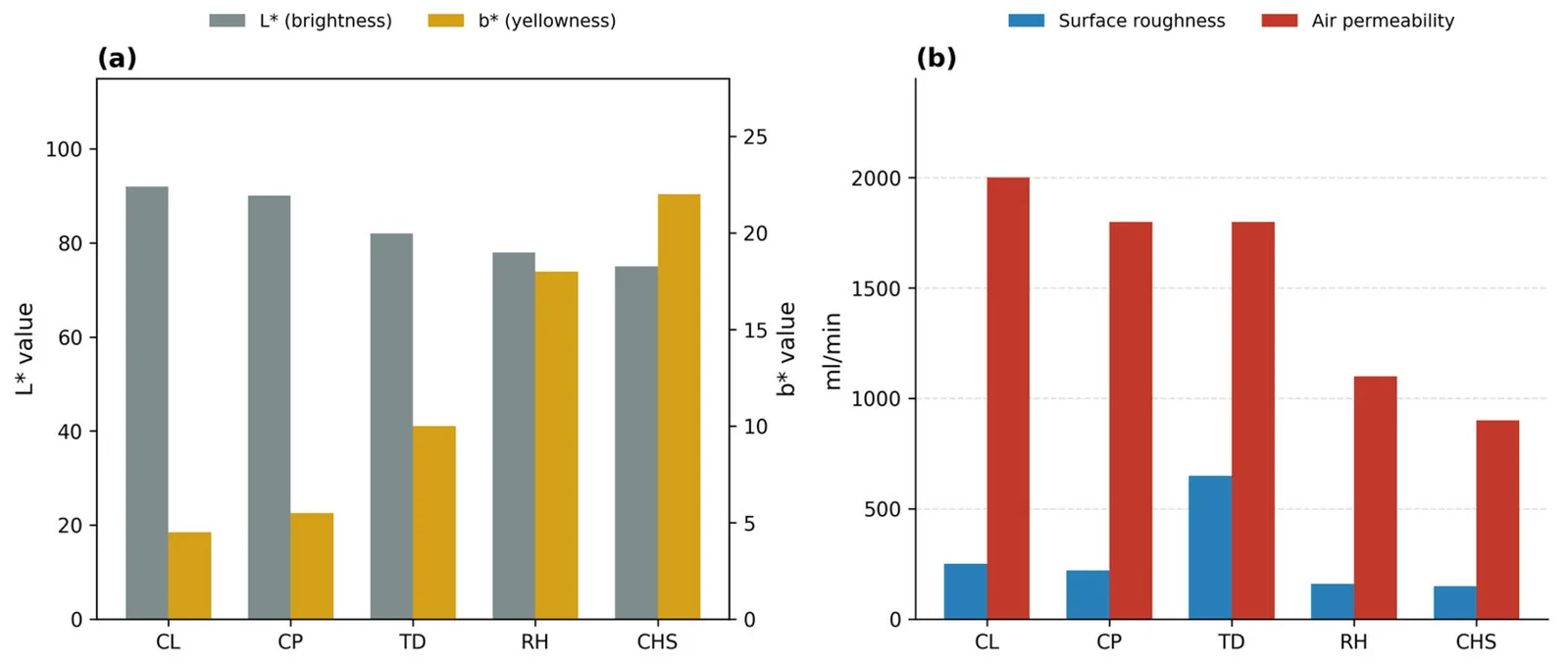
Comparative summary of paper optical and surface properties across all five raw-material series: (**a**) CIE L* (brightness) and b* (yellowness) coordinates (consolidated from Table 13); (**b**) surface roughness and air permeability (consolidated from Table 14).

The highest brightness and lowest yellowness were measured for CL (L* = 92.0; b* = +4.5) and CP (Figure 13a) (L* = 90.0; b* = +5.5); this confirms that both raw materials have a high-purity, lignin-poor cellulosic structure. CHS, of agricultural-waste origin, exhibited the lowest brightness (L* = 75.0) and the highest yellowness (b* = +22.0), indicating the presence of residual lignin and chromophoric groups.

The lowest surface roughness and air permeability values were measured for the CHS sample (150 mL/min; 900 mL/min) (Figure 13b), which has the densest and most closed fibre network. TD, in contrast, exhibited the highest surface roughness (650 mL/min), while CL exhibited the highest air permeability (2000 mL/min), indicating a loose, bulky and porous structure. These results are consistent with the earlier density and mechanical test findings.

### 3.6. Structural and Morphological Characterisation

#### 3.6.1. FTIR Analysis

**Figure 14:**
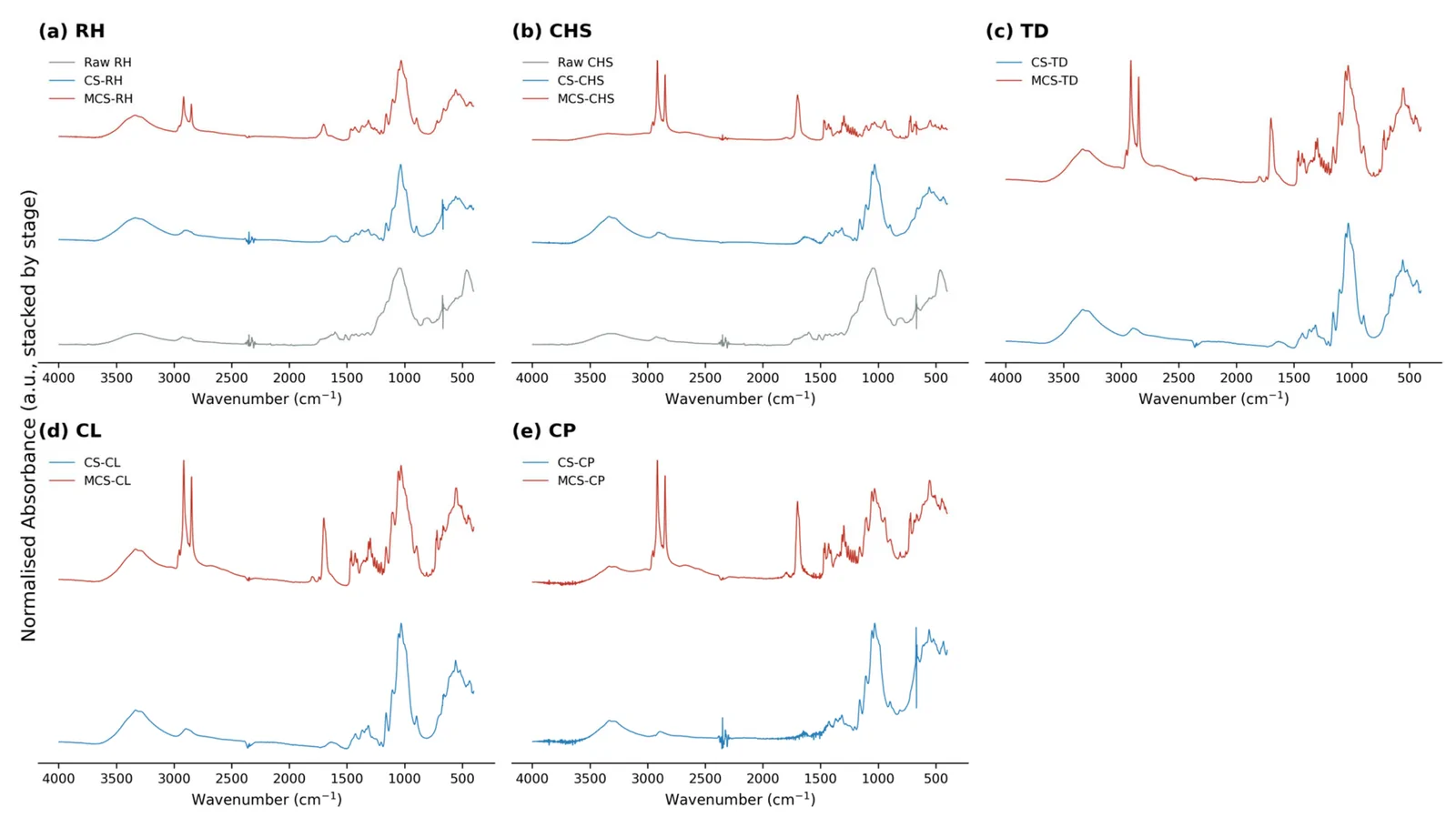
FTIR spectra of all five raw-material series at each available processing stage, stacked by stage: (**a**) RH (raw, CS and MCS); (**b**) CHS (raw, CS and MCS); (**c**) TD (CS and MCS); (**d**) CL (CS and MCS); (**e**) CP (CS and MCS). The new ester carbonyl (C=O) band at ~1700–1730 cm^−1^, present only in the MCS spectra, is visible in every panel.

FTIR analyses were used as a fundamental tool for chemically verifying the production and modification processes; spectra for all five raw-material series, compared at each available processing stage, are presented together in Figure 14. In the RH and CHS series, for which a raw-material spectrum was also available, the broad -OH band at ~3320–3350 cm^−1^ and the complex peaks in the fingerprint region of the raw spectra reflect the typical characteristics of lignocellulosic biomass; in the corresponding CS spectra obtained after delignification, the marked reduction in the peaks at ~1730 cm^−1^ and ~1500–1600 cm^−1^, attributed respectively to the acetyl and uronic ester (C=O) groups of hemicellulose and to the aromatic skeletal vibrations of lignin [30], demonstrates the success of the purification process, while the intense C-O-C peak at ~1024 cm^−1^ confirms the cellulosic character common to all five CS spectra. In every one of the five MCS spectra, a new, sharp peak appears at approximately 1700–1730 cm^−1^ that is absent from the corresponding CS (and, where available, raw) spectrum; this peak belongs to the ester carbonyl (C=O) group formed as a result of the esterification reaction between the hydroxyl groups of cellulose and stearoyl chloride. In addition, the marked increase in the intensity of the C-H stretching peaks at ~2850–2920 cm^−1^ in every MCS spectrum confirms the presence of the long (C18) hydrophobic hydrocarbon chain successfully grafted onto the cellulose surface. These spectral changes were observed consistently across all five raw-material sources and demonstrate that the stearoyl chloride modification was chemically achieved with success, independent of raw material source.

#### 3.6.2. SEM Analysis

**Figure 15:**
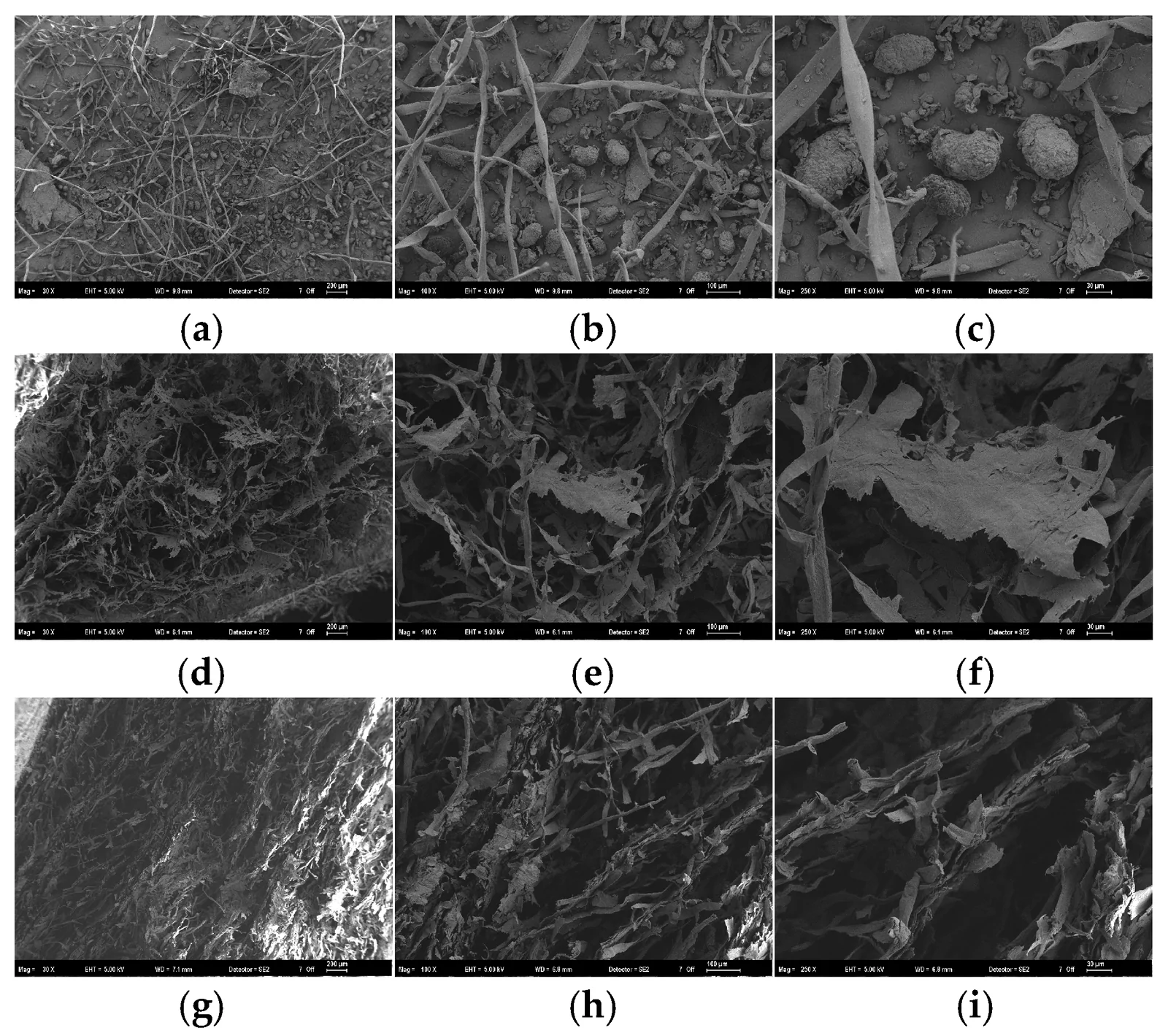
SEM images of CHS samples, labelled (**a**–**i**) in row-major order—row 1 (**a**–**c**): raw CHS; row 2 (**d**–**f**): CS-CHS; row 3 (**g**–**i**): MCS-CHS; columns: 30×, 100×, 250× magnification, respectively.

**Figure 16:**
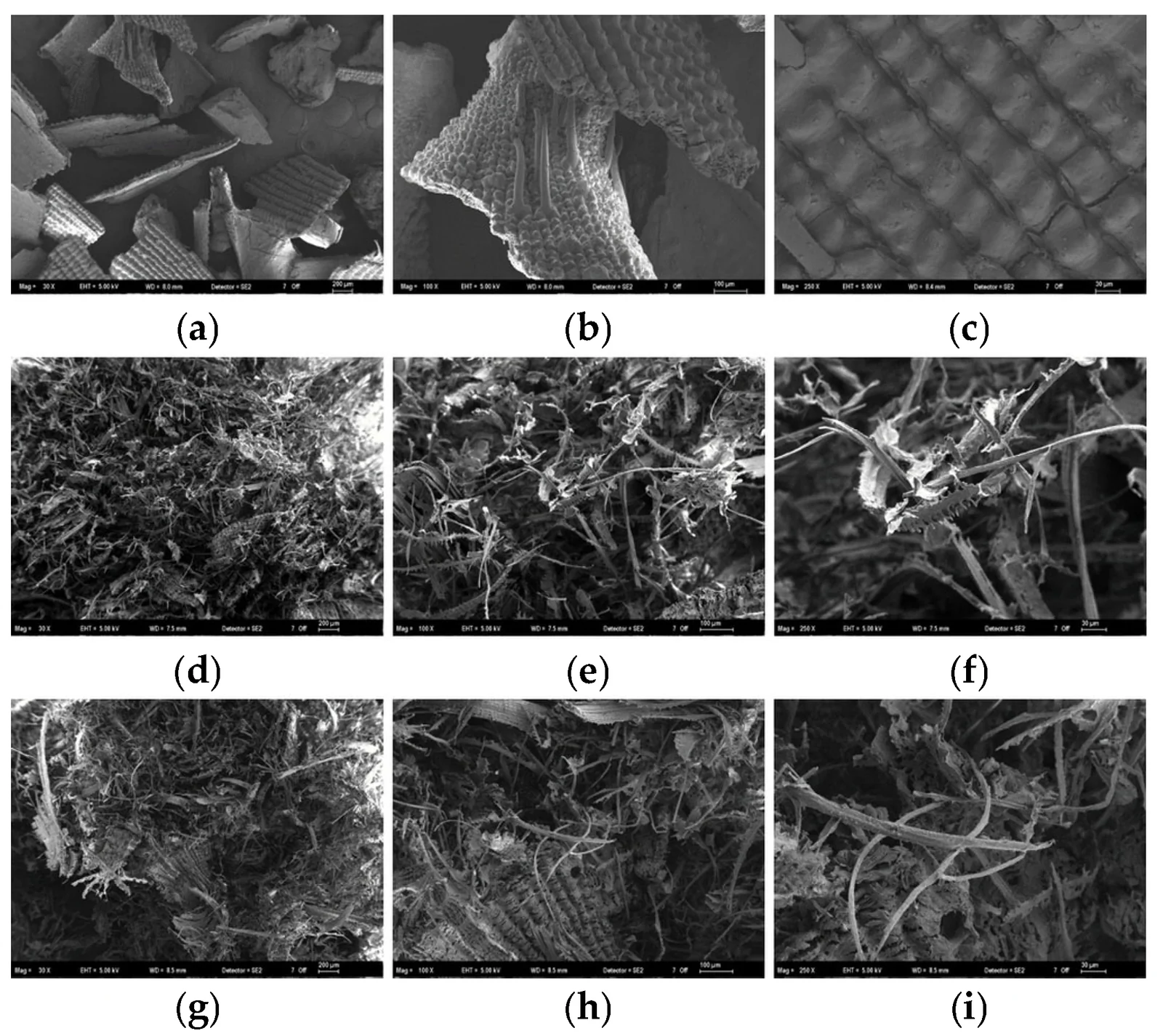
SEM images of RH samples, labelled (**a**–**i**) in row-major order—row 1 (**a**–**c**): raw RH; row 2 (**d**–**f**): CS-RH; row 3 (**g**–**i**): MCS-RH; columns: 30×, 100×, 250× magnification, respectively.

**Figure 17:**
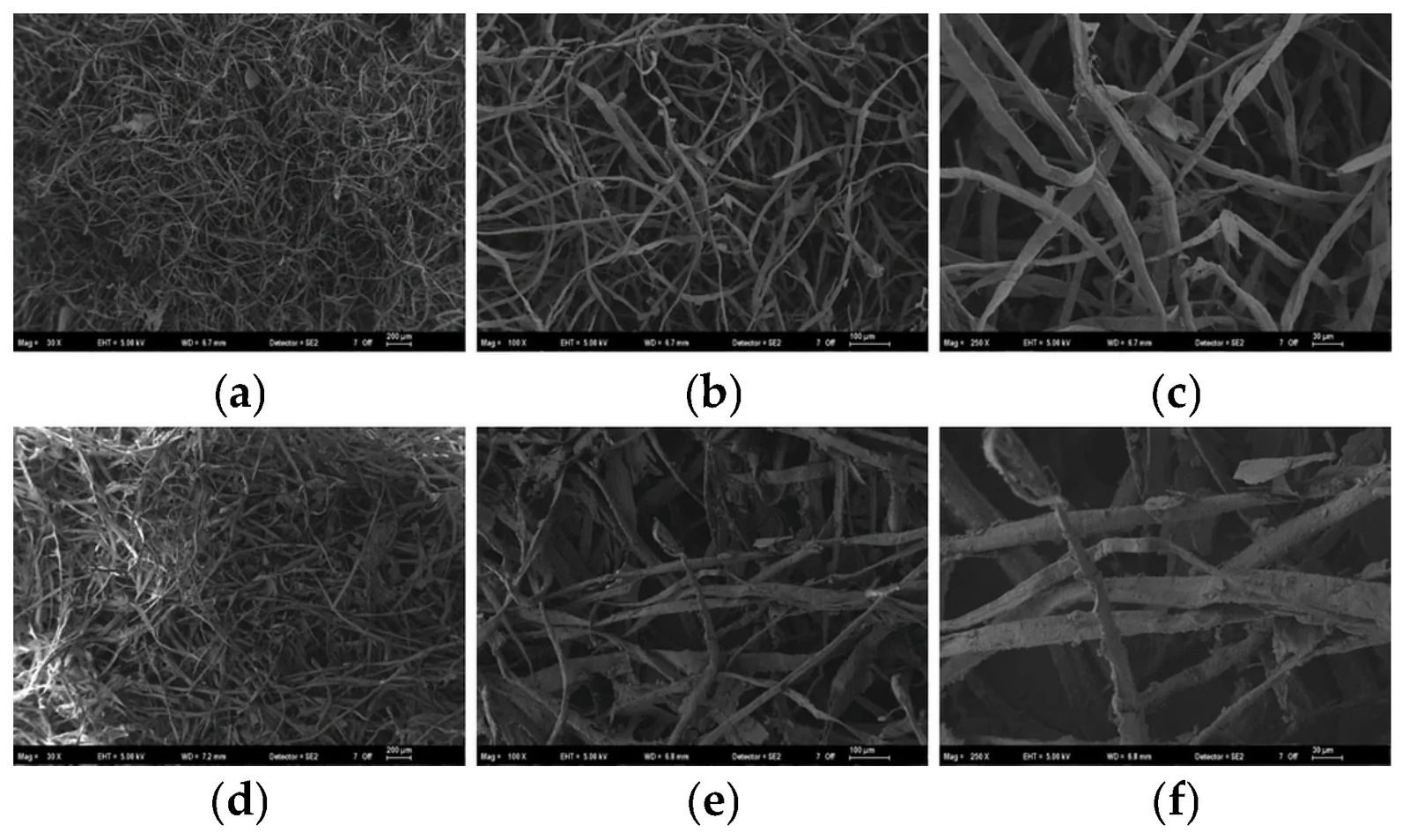
SEM images of CP samples, labelled (**a**–**f**) in row-major order—row 1 (**a**–**c**): CS-CP; row 2 (**d**–**f**): MCS-CP; columns: 30×, 100×, 250× magnification, respectively.

**Figure 18:**
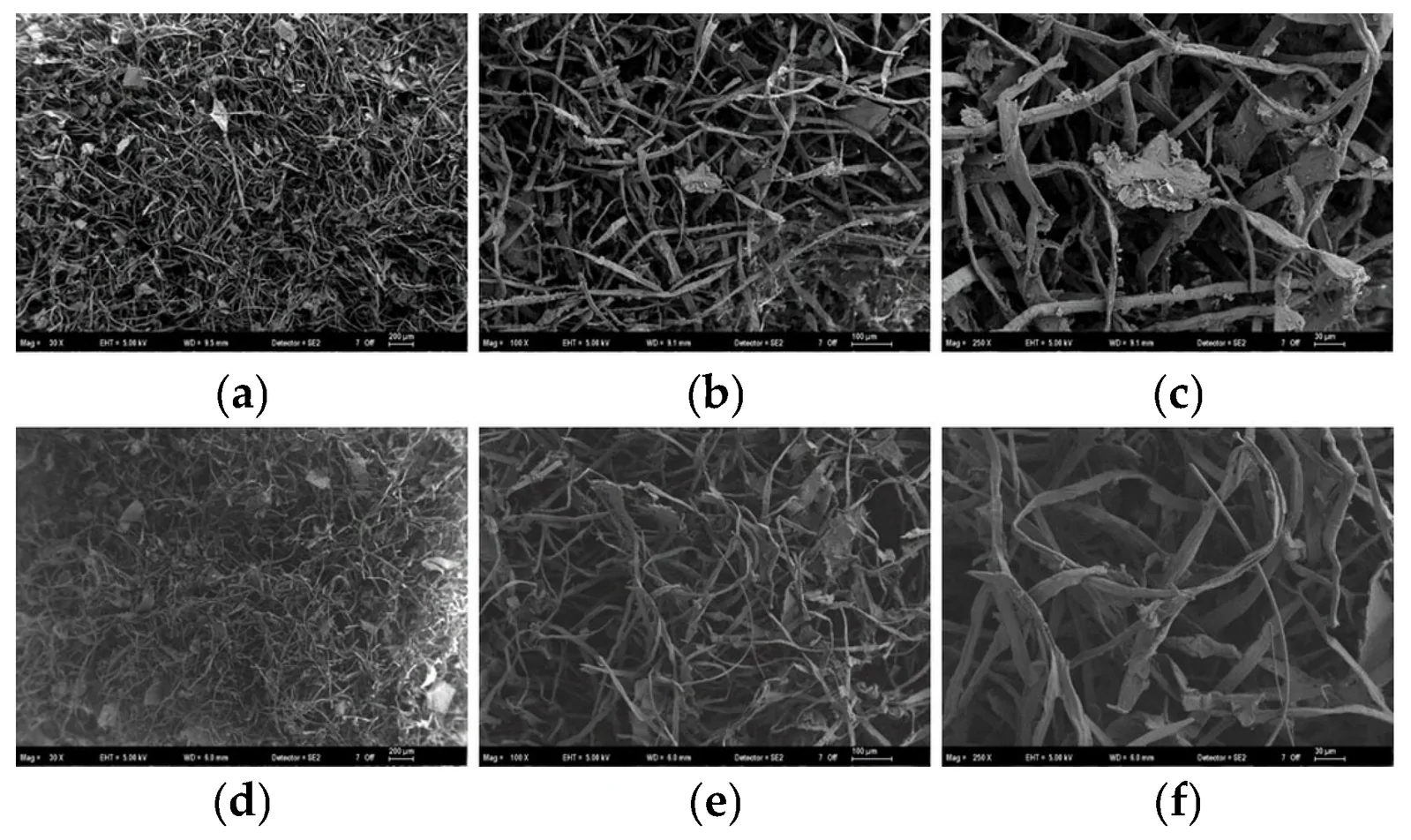
SEM images of CL samples, labelled (**a**–**f**) in row-major order—row 1 (**a**–**c**): CS-CL; row 2 (**d**–**f**): MCS-CL; columns: 30×, 100×, 250× magnification, respectively.

SEM examinations visually confirmed the microstructural evolution from raw material to modified sponge. In the chestnut shell (CHS) series presented in Figure 15, heterogeneous, individual and woody lignocellulosic fibres are observed in the raw material, whereas after delignification the fibres in the cellulose sponge (CS-CHS) acquire a more flexible, individual structure and randomly come together to form a highly porous, three-dimensional network. After modification (MCS-CHS), this porous architecture is largely preserved, and the high surface area and interconnected pore structure form the microstructural basis of the high adsorption capacity observed in the study. A similar evolution was observed in the rice husk (RH) series (Figure 16); similar patterns were also observed in the chemical pulp (CP) and cotton lint (CL) series (Figure 17 and Figure 18). The characteristic rough, silica-rich cuticle structure of raw RH largely disappeared after delignification, giving way to a porous network of loose, entangled cellulose fibres, and this fibrous-porous architecture was preserved after modification.

#### 3.6.3. XRD Analysis

**Figure 19:**
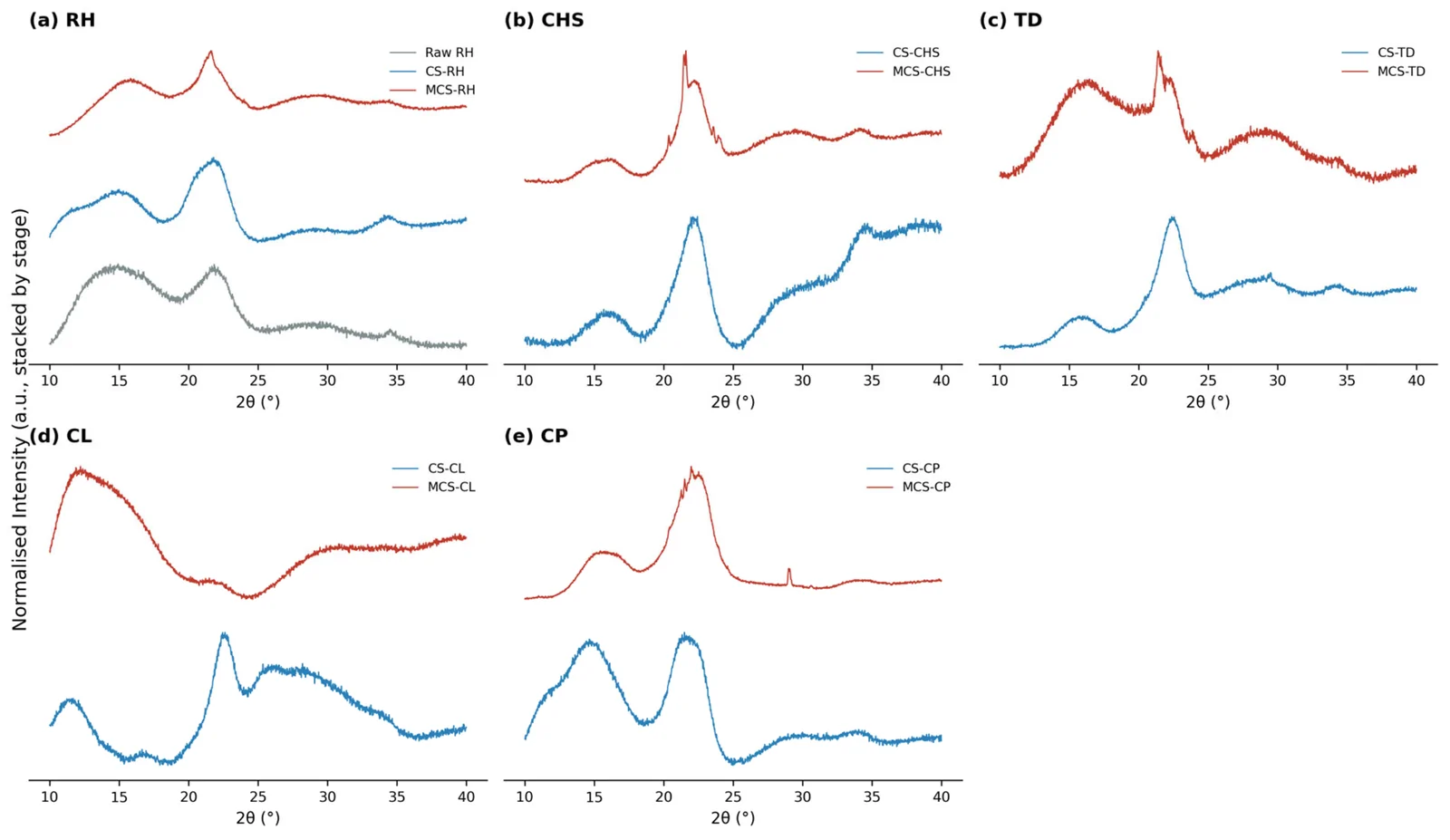
XRD diffractograms (2θ = 10–40°) for all five raw-material series, stacked by processing stage: (**a**) RH (raw, CS and MCS); (**b**) CHS; (**c**) TD; (**d**) CL; (**e**) CP (CS and MCS only for (**b**–**e**), as no separate raw-material XRD scan was taken for these series). Note the anomalous new low-angle peak (~12.2°) unique to MCS-CL in panel (**d**).

XRD analyses were performed on the raw material, cellulose sponge (CS) and modified sponge (MCS) stages of the five raw-material series (raw material available for RH only; see Methods); the resulting diffractograms are compared in Figure 19. In four of the five series (RH, CHS, TD and CP), a consistent pattern was observed: characteristic cellulose I peaks (2θ ≈ 16.4° and 22.6°) [31] were present at every stage, these peaks became sharper and more pronounced after delignification (CS), and a new shoulder near 2θ ≈ 19.2° appeared after stearoyl chloride modification (MCS) without displacing the main crystalline reflections (Table 15). The corresponding Segal crystallinity index [31] increased from the raw material to CS for RH, and was higher for MCS than for CS in the CHS and CP series, consistent with the removal of amorphous lignin and hemicellulose during purification and, for MCS, with the additional ordering contributed by the grafted side chains (Table 16).

The cotton-lint series (CL) deviated markedly from this pattern (Figure 19d). While CS-CL showed the expected cellulose I reflections, MCS-CL exhibited a new, dominant low-angle peak at 2θ ≈ 12.2° whose intensity exceeded that of the residual cellulose 002 reflection; as a result, the standard Segal index could not be meaningfully applied to this sample (Table 16). Because CL is the purest, most crystalline of the five substrates (Table 1) and MCS-CL was also the only sample to show a distinct, high-temperature secondary DTG peak at 433 °C (Section 3.6.4), this new reflection is attributed to ordered, lamellar packing of the grafted C18 stearate side chains on the CL fibre surface, providing direct diffraction-based support for the side-chain crystallisation hypothesis proposed to explain the anomalous thermal stability of MCS-CL, rather than reflecting a change in the underlying cellulose lattice itself. Representative characteristic peak positions for the general RH/CHS/TD/CP pattern are summarised in Table 15.

#### 3.6.4. TGA Analysis

**Figure 20:**
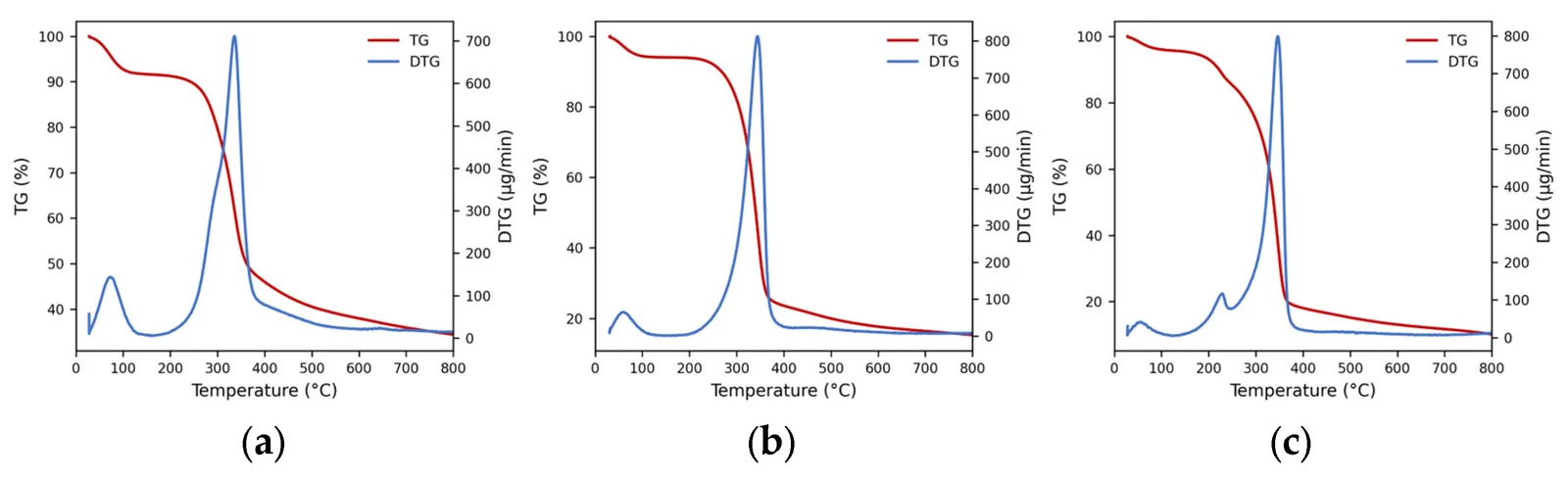
TG and DTG curves of the RH series: (**a**) raw RH, (**b**) CS-RH, (**c**) MCS-RH.

**Figure 21:**
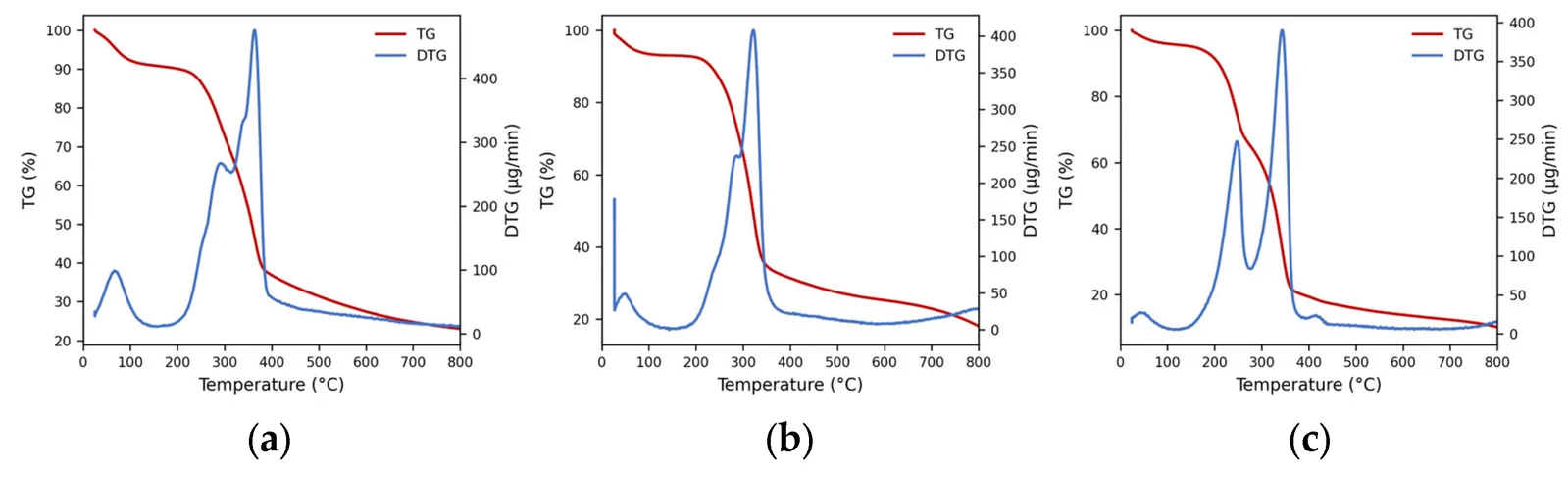
TG and DTG curves of the CHS series: (**a**) raw CHS, (**b**) CS-CHS, (**c**) MCS-CHS.

**Figure 22:**
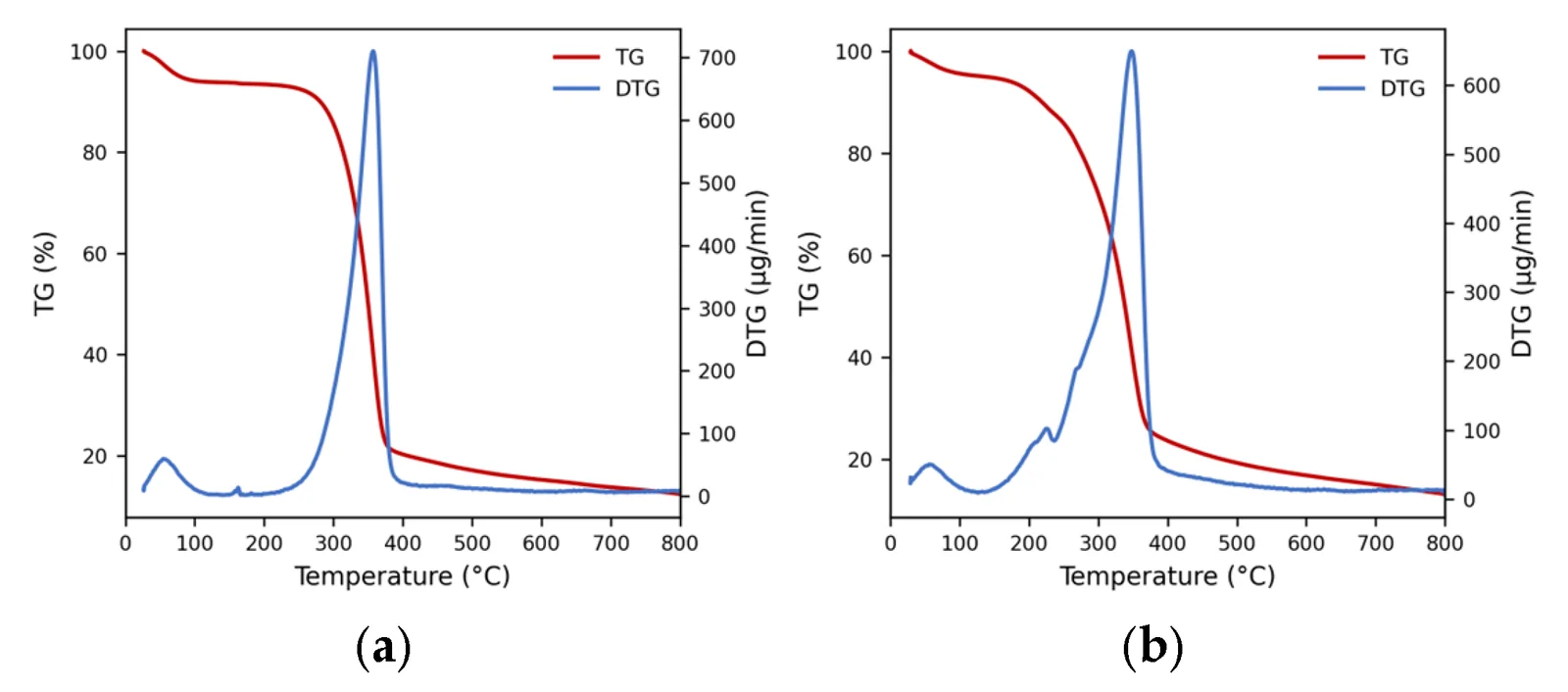
TG and DTG curves of the TD series: (**a**) CS-TD, (**b**) MCS-TD.

**Figure 23:**
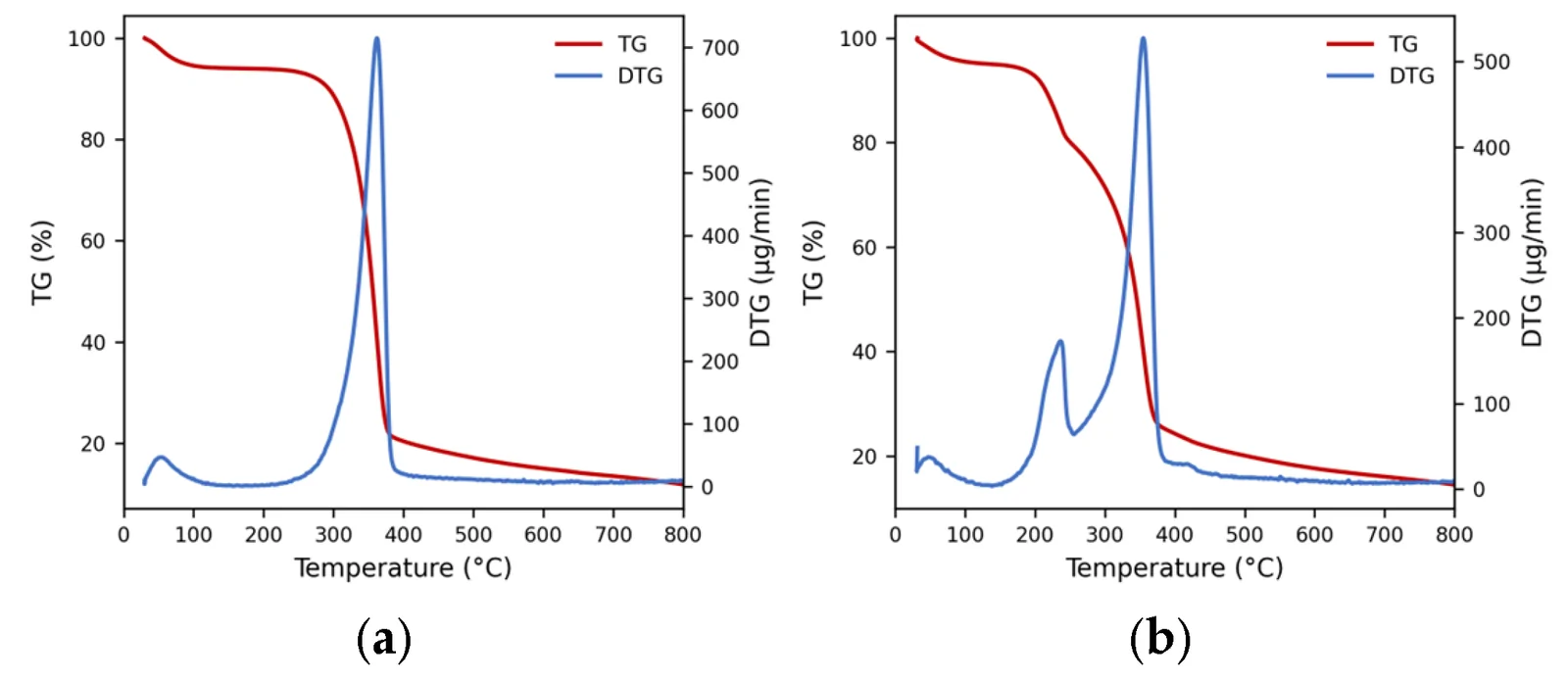
TG and DTG curves of the CP series: (**a**) CS-CP, (**b**) MCS-CP.

**Figure 24:**
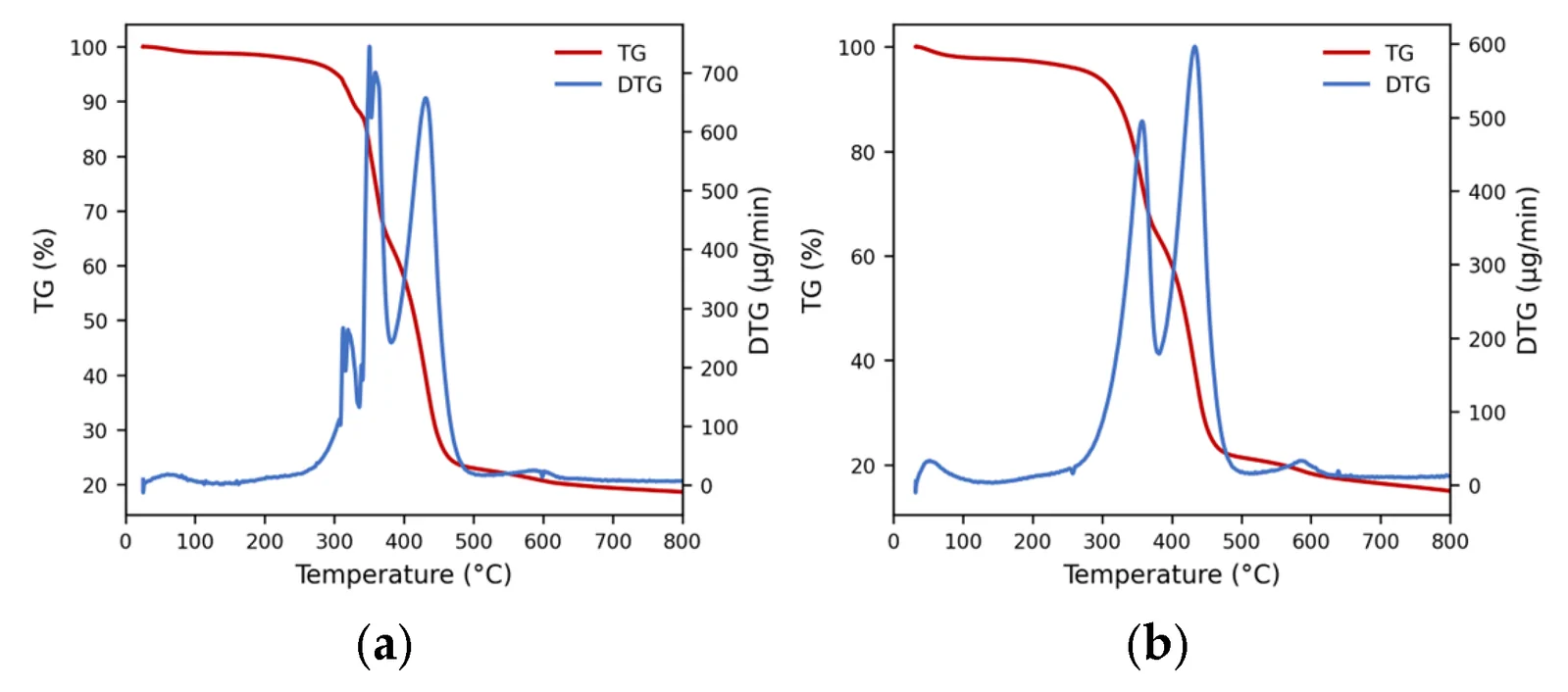
TG and DTG curves of the CL series: (**a**) CS-CL, (**b**) MCS-CL.

Simultaneous TGA/DTA analyses revealed that all samples exhibited a three-stage thermal decomposition profile: an initial moisture-loss stage below approximately 150 °C, a main organic decomposition stage, and a slow carbonisation stage extending to 800 °C (Table 17; Figure 20, Figure 21, Figure 22 and Figure 23). In the main decomposition stage, the onset temperature and the DTG peak (maximum mass-loss rate, Tmax) both shifted to considerably lower values after cellulose extraction and, in most series, after chemical modification: for the RH series, the onset fell from 220 °C (raw RH and CS-RH) to 216 °C (MCS-RH), while for the CHS, TD and CP series the onset dropped more markedly, from 200 to 250 °C in the unmodified samples to 155 °C in the modified (MCS) samples. The double DTG peaks observed in the raw biomasses (RH, CHS) and in several CS samples (e.g., 300 °C and 370 °C for CHS; 270 °C and 330 °C for CS-CHS) were attributed to the separate decomposition of hemicellulose and cellulose fractions [32]; after delignification and, more distinctly, after modification, these merged into fewer, more clearly defined peaks, consistent with a more homogeneous cellulosic decomposition profile. Stearoyl chloride modification markedly reduced the residual mass (char yield) at 800 °C in most sample series (e.g., from 15% for CS-RH to 10% for MCS-RH; from 17% for CS-CHS to 9% for MCS-CHS), showing that the organic stearoyl mass added to the structure largely decomposed and volatilised by the analysis temperature under the nitrogen atmosphere used; this provides quantitative evidence, alongside the FTIR results, that the chemical modification took place. Because the analysis was carried out under a N_2_ (pyrolytic, non-oxidative) atmosphere rather than in air, this 800 °C residue reflects carbonised char plus any true inorganic ash, rather than ash alone. The TD and CP series showed a smaller or even slightly reversed residue change (12% to 13% for TD; 11% to 14% for CP), which may reflect a smaller relative mass contribution of the grafted stearoyl layer in these already low-ash, high-purity fibres. Raw TGA data for both the unmodified and modified CL samples were additionally evaluated (Figure 24). The unmodified CS-CL sample showed a markedly low moisture loss of 1.2% (substantially lower than all other raw materials), a decomposition onset at 250 °C, a DTG peak at 350 °C, and a residue of 19% at 800 °C. The low moisture content is consistent with the cotton-derived CL fibre being considerably less hygroscopic and structurally more homogeneous than the other lignocellulosic raw materials. The modified MCS-CL sample, in contrast, showed its main decomposition process split into two distinct DTG peaks (350 °C and 433 °C); this second, dominant peak at 433 °C is thought to arise from the decomposition of the grafted stearoyl chains occurring at a somewhat higher temperature, separate from that of the cellulosic backbone. This behaviour finds support in the literature on long-chain cellulose esters: Jandura et al. [33] reported that cellulose fibres partially esterified with long-chain organic acids, including stearic acid, exhibit a two-step degradation process, likely governed by crosslinking reactions occurring during thermal decomposition, closely mirroring the double-peak profile observed here for MCS-CL. In addition, Huang [34] showed that fully stearoylated cellulose (cellulose tri-stearate, CTs) exhibits markedly higher thermal stability than unmodified microcrystalline cellulose (DTG peak: 371 °C for CTs versus 344.6 °C for MCC), an improvement attributed to the regular, ordered arrangement of the long C18 stearate side chains on the cellulose backbone (side-chain crystallisation). In light of this literature, the unusually high and dominant peak at 433 °C observed for MCS-CL may reflect a more extensive and orderly grafting of stearoyl groups onto this particularly high-purity fibre (α-cellulose 93.5%), producing a similar crystalline side-chain arrangement to that reported by Huang [34] and thereby enhancing thermal stability beyond that observed for the other, less pure MCS series. The residue of MCS-CL at 800 °C (15%) was lower than that of CS-CL (19%), consistent with the trend observed for the other raw material series, in which modification largely removes the added organic stearoyl mass by the analysis temperature.

The XRD and TGA/DTG results are consistent with one another, though the relationship is not a simple monotonic one across all series. The Segal crystallinity index (CrI, Table 16) increased from the raw material to the cellulose sponge (CS) stage in the one series where a raw-material scan was available (RH: 18.1% to 42.7%), consistent with the removal of amorphous lignin and hemicellulose during purification. Across the modified (MCS) samples, CrI ranged from 22.6% (MCS-TD) to 71.7% (MCS-CHS), and this ranking did not translate into a simple, proportional ranking of DTG peak (Tmax) temperatures (Table 17): MCS-CHS, despite having the highest CrI among the conventional series, had a Tmax of only 340 °C, similar to the much less crystalline MCS-TD (CrI 22.6%, Tmax 340 °C), indicating that factors other than bulk cellulose crystallinity, such as residual inorganic content and the extent of stearoyl substitution, also govern thermal stability in this system. The clearest and most consistent link between the two techniques concerns the cotton-lint (CL) series specifically: MCS-CL was the only sample for which both the main-decomposition onset (250 °C, versus 155–216 °C for the other MCS series) and the DTG peak (433 °C) remained markedly elevated, and it was also the only sample whose XRD pattern showed a new, dominant reflection unrelated to the native cellulose lattice (2θ ≈ 12.2°, Section 3.6.3, Figure 19d). Taken together, these two independent observations support the interpretation that an ordered, lamellar packing of the grafted C18 stearate side chains on the CL fibre surface, rather than the crystallinity of the underlying cellulose backbone, is primarily responsible for the distinctive thermal behaviour of MCS-CL.

**Figure 25:**
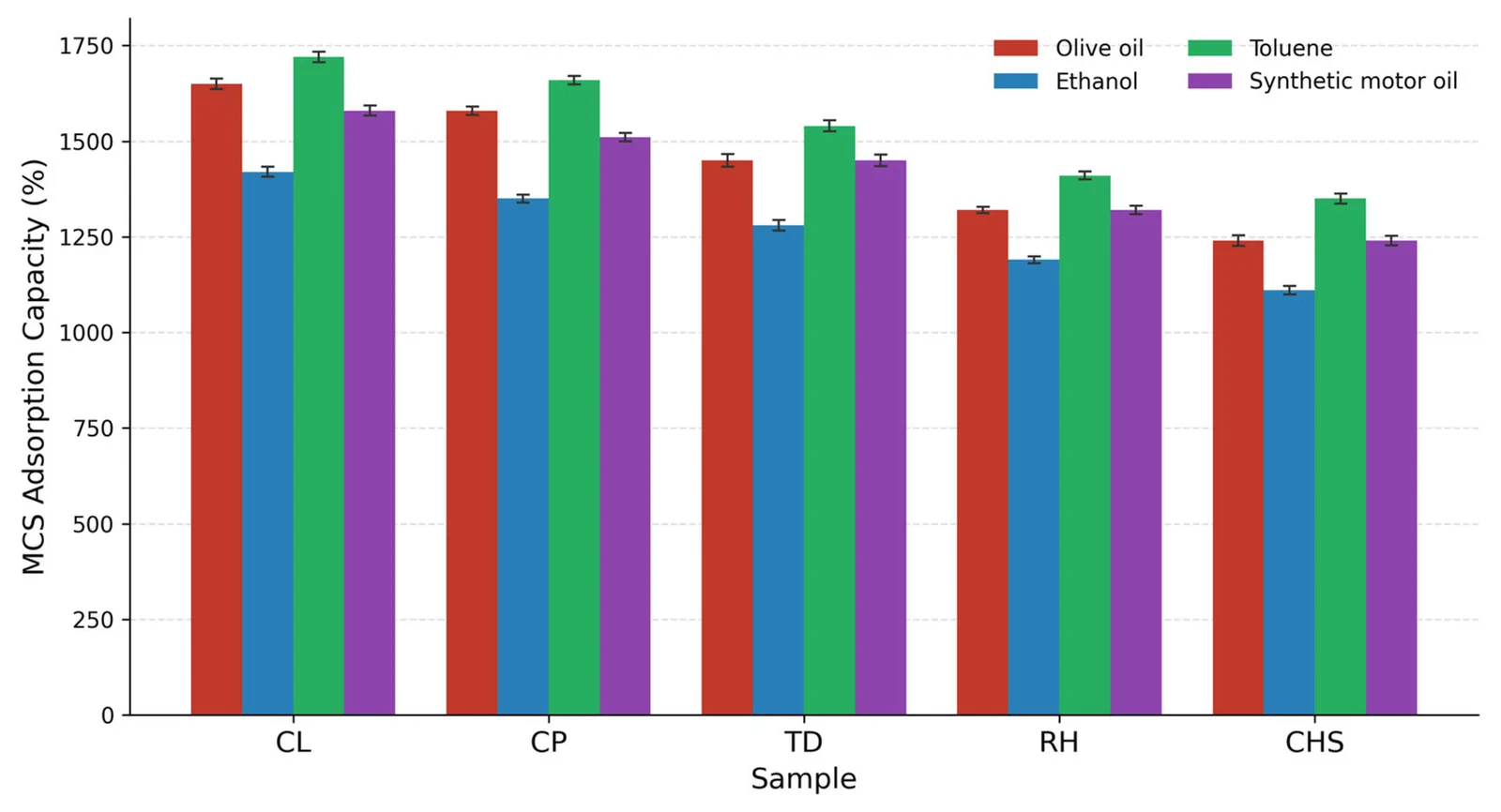
Comparative summary of MCS adsorption capacity for olive oil, ethanol, toluene and synthetic motor oil across all five raw-material series (consolidated from Table 4, Table 5, Table 6 and Table 7; error bars show ±1 SD of three replicate measurements).

**Figure 26:**
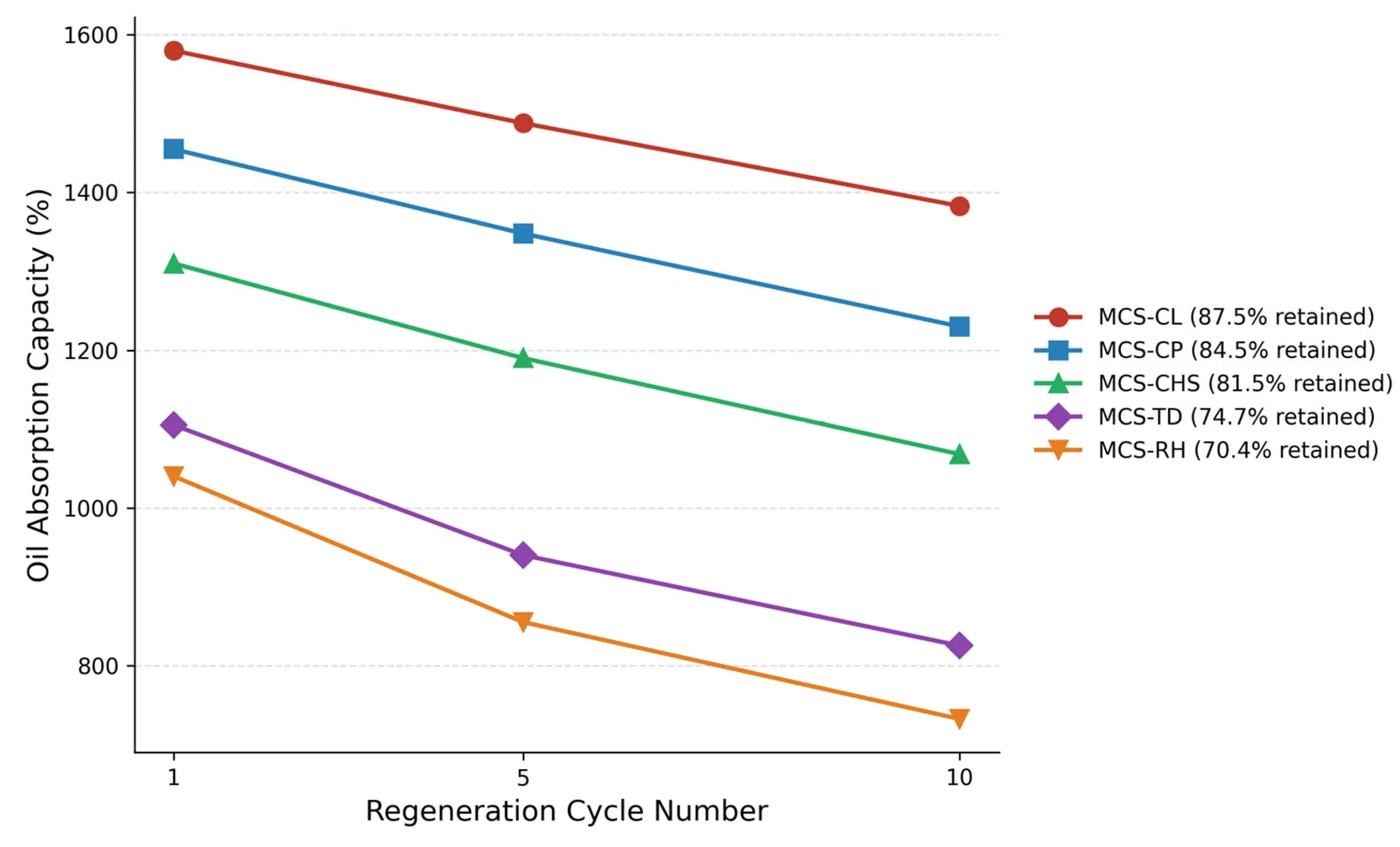
Decline in oil absorption capacity of the five MCS series over ten adsorption-regeneration cycles, with final capacity retention ratios indicated in the legend (consolidated from Table 8).

This interpretation is consistent with the broader literature on long-chain fatty acid cellulose esters. Cellulose esters grafted with C8-C18 acyl chains are known to adopt a layered structure in which the cellulosic backbones lie in one plane while the fully extended side chains project perpendicular to it, and once the chain length exceeds approximately 12 carbons a fraction of these side chains crystallise into their own, separate ordered packing, distinct from the cellulose I lattice [35]. Because this side-chain crystallisation is known to depend strongly on the degree of substitution (DS), appearing only once DS is sufficiently high and spatially uniform [36], and comparable new low-angle XRD reflections attributed to the crystallisation of grafted long-chain fatty-acid substituents have been reported specifically for esterified cotton cellulose [37] and for other fibrous long-chain cellulose esters [38], the appearance of this feature in only one of the five raw-material series examined here is plausible rather than anomalous. Among the five substrates, CL combines the highest α-cellulose content (93.5%) with the lowest ash content (5.8%) (Table 1), and is therefore the most chemically homogeneous and accessible of the five fibres; we propose that this favoured a higher and more spatially uniform degree of stearoyl substitution on CL than on the other, more heterogeneous or impurity-laden substrates, allowing the grafted chains to nucleate a coherent, diffracting crystalline phase (Figure 25 and Figure 26).

### 3.7. Overall Assessment of the Study

When all the findings obtained are considered together, it is seen that the fundamental variable determining the performance of the modified cellulose sponges produced is the chemical purity of the raw material (in particular the α-cellulose content and the lignin/extractive ratio). Sponges produced from high-purity sources such as cotton lint (CL) and chemical pulp (CP) consistently exhibited the highest contact angle, the lowest residual water absorption ratio and the highest oil/solvent adsorption capacity. Unprocessed agricultural wastes such as rice husk (RH) and chestnut shell (CHS), in contrast, showed a more modest hydrophobic transformation because the lignin, extractive and inorganic (silica/ash) fractions in their structure sterically hindered the modification reaction. Interestingly, these same impurities created a “filler” effect in the paper matrix, increasing the tensile strength of RH- and CHS-derived samples but reducing their flexibility (elongation percentage); this shows that raw material selection involves a trade-off between sponge performance and mechanical durability.

## 4. Conclusions

In this study, three-dimensional porous sponges were produced by freeze-drying cellulose fibres obtained from five different lignocellulosic wastes (rice husk, chestnut shell, tissue dust, cotton lint and chemical pulp), and these sponges were rendered hydrophobic/oleophilic through an esterification reaction with stearoyl chloride. Chemical characterisation of the raw materials revealed that chemical pulp offers the purest cellulosic structure, while the agricultural wastes (rice husk, chestnut shell) exhibit a more heterogeneous profile with high hemicellulose and lignin content.

The effectiveness of the chemical modification was quantitatively demonstrated by the increase in contact angle from 0° (superhydrophilic) to 115–136.5° (hydrophobic) and the dramatic decrease in water absorption capacity. The new ester carbonyl peak observed at ~1701–1730 cm^−1^ in the FTIR spectra constitutes the chemical evidence of this transformation. The modified sponges were able to adsorb oil and organic solvents at ratios ranging from 12 to 17 times their own dry weight; a large part of this capacity was retained (70.4–87.5%) even after ten adsorption-regeneration cycles. The findings obtained show that raw material selection directly affects both initial performance and cyclic durability, and that sponges produced from long, processed fibres (cotton lint, chemical pulp, chestnut shell) are mechanically more durable.

Regarding end-of-life management, incineration of the oil-saturated sponges recovers the calorific value of both the cellulosic matrix and the sorbed hydrocarbons but is not, in itself, an environmentally preferable outcome; alternative routes such as controlled pyrolysis with energy and biochar recovery, or solvent-based oil recovery followed by composting or anaerobic digestion of the regenerated cellulosic material, would be more consistent with a circular-economy framing and merit dedicated investigation.

In conclusion, modified cellulose sponges produced from lignocellulosic wastes offer an effective and sustainable alternative to conventional methods, as an environmentally friendly, waste-derived, high-performance oil-sorbent material with promising but as-yet-unquantified cost advantages, for cleaning up oil spills and treating industrial oily wastewater. In future studies, it is recommended that durability tests be carried out over a wide pH range and under UV irradiation exposure in order to evaluate the long-term persistence of the modification; that process optimisation studies be conducted to increase the industrial-scale economic feasibility of the synthesis process; and that environmentally sustainable disposal methods be investigated for sponges that have reached the end of their service life. In addition, and beyond the scope of the present comparative screening study, we recommend that future work quantify the time-dependent adsorption kinetics of the sponges; evaluate the mechanical durability of the modified sponge (aerogel) form itself, rather than the paper-based proxy used here, under repeated cyclic compression; assess performance against surfactant-stabilised oil-in-water emulsions and real or field-collected oily wastewater, rather than pure oils and solvents alone; and characterise the residual oil and water quality of the separated phases against applicable environmental discharge standards. These additional experimental campaigns represent clear and valuable next steps for translating this material from a laboratory-scale comparative study towards a field-ready technology.

## Figures and Tables

**Table 1:** Chemical composition of the lignocellulosic raw materials used.

Raw Material	Moisture (%)	Ash (%)	Total Extractives (%)	1% NaOH Sol. (%)	Hot Water Sol. (%)	Total Lignin (%)	Holocellulose (%)	α-Cellulose (%)
RH	9.3	16.2	3.45	7.9	0.30	21.30	55.81	40.47
CHS	8.3	2.1	22.00	18.0	26.0	33.50	35.00	21.00
TD	7.5	11.5	1.00	15.5	4.5	3.80	95.00	86.50
CL	7.8	5.8	1.50	12.8	3.1	2.50	95.80	93.50
CP	6.5	0.8	0.50	6.2	1.8	0.80	98.50	90.20

**Table 2:** Mean static contact-angle values of MCS samples.

Sample Code	Mean Static Contact Angle (θ)
MCS-CL	136.5°
MCS-TD	127.5°
MCS-RH	125.26°
MCS-CHS	122.64°
MCS-CP	115.0°

**Table 3:** Water absorption capacities of CS and MCS samples.

Sample Code	CS Water Absorption (%)	MCS Water Absorption (%)
CL	1450 ± 11	26 ± 1
CP	1380 ± 11	31 ± 2
TD	1310 ± 16	54 ± 3
RH	1250 ± 9	48 ± 2
CHS	1180 ± 12	35 ± 3

**Table 4:** Olive oil adsorption capacities of CS and MCS samples.

Sample Code	CS Oil Absorption (%)	MCS Oil Absorption (%)
CL	450 ± 8	1650 ± 14
CP	420 ± 5	1580 ± 11
TD	480 ± 8	1450 ± 16
RH	550 ± 5	1320 ± 9
CHS	580 ± 7	1240 ± 13

**Table 5:** Ethanol adsorption capacities of CS and MCS samples.

Sample Code	CS Ethanol Absorption (%)	MCS Ethanol Absorption (%)
CL	1050 ± 10	1420 ± 13
CP	1010 ± 8	1350 ± 10
TD	1080 ± 13	1280 ± 14
RH	1150 ± 7	1190 ± 9
CHS	1180 ± 10	1110 ± 11

**Table 6:** Toluene adsorption capacities of CS and MCS samples.

Sample Code	CS Toluene Absorption (%)	MCS Toluene Absorption (%)
CL	180 ± 3	1720 ± 14
CP	160 ± 3	1660 ± 11
TD	190 ± 4	1540 ± 15
RH	240 ± 4	1410 ± 11
CHS	220 ± 4	1350 ± 13

**Table 7:** Synthetic motor oil adsorption capacities of CS and MCS samples.

Sample Code	CS Synthetic Oil Absorption (%)	MCS Synthetic Oil Absorption (%)
CL	170 ± 3	1580 ± 13
CP	150 ± 3	1510 ± 11
TD	180 ± 5	1450 ± 15
RH	210 ± 4	1320 ± 11
CHS	200 ± 4	1240 ± 13

**Table 8:** Comparison of the oil absorption capacities of MCS samples after ten cycles (%).

Cycle Number	MCS-CL (%)	MCS-CP (%)	MCS-CHS (%)	MCS-TD (%)	MCS-RH (%)
1	1580	1455	1310	1105	1040
5	1488	1348	1190	940	855
10	1383	1230	1068	825	732
Capacity Retention Ratio (%)	87.5	84.5	81.5	74.7	70.4

**Table 9:** Schopper-Riegler freeness (°SR) values of the different cellulosic pulps.

Sample Code	Schopper-Riegler Freeness (°SR)
CHS	28
CL	22
CP	16
TD	14
RH	12

**Table 10:** pH values of the different cellulosic pulps.

Sample Code	pH Value
CL	6.5
CP	6.8
TD	7.1
RH	7.6
CHS	8.0

**Table 11:** Grammage, thickness, density and bulk values of the papers produced from the different cellulosic pulps.

Sample Code	Grammage (g/m^2^)	Thickness (µm)	Density (g/cm^3^)	Bulk (cm^3^/g)
CL	65.2 ± 0.7	141 ± 3	0.46 ± 0.02	2.16 ± 0.04
CP	64.8 ± 0.6	126 ± 2	0.51 ± 0.03	1.95 ± 0.03
TD	65.5 ± 0.8	116 ± 4	0.56 ± 0.02	1.77 ± 0.04
RH	64.9 ± 0.4	98 ± 3	0.66 ± 0.04	1.51 ± 0.02
CHS	65.1 ± 1.0	93 ± 4	0.70 ± 0.03	1.43 ± 0.03

**Table 12:** Tensile test results of the papers produced from the different cellulosic pulps.

Sample Code	Tensile Strength (kN/m)	Breaking Length (km)	Elongation (%)
CL	2.48 ± 0.04	3.8 ± 0.2	3.5 ± 0.3
CP	2.72 ± 0.04	4.2 ± 0.3	3.1 ± 0.2
TD	2.29 ± 0.05	3.5 ± 0.2	2.4 ± 0.1
RH	2.92 ± 0.03	4.5 ± 0.4	1.8 ± 0.2
CHS	3.13 ± 0.06	4.8 ± 0.3	1.6 ± 0.4

**Table 13:** CIE L*a*b* colour coordinate values of the papers produced from the different cellulosic pulps.

Sample Code	L* Value (Brightness)	b* Value (Yellowness/Blueness)
CL	92.0	+4.5
CP	90.0	+5.5
TD	82.0	+10.0
RH	78.0	+18.0
CHS	75.0	+22.0

**Table 14:** Surface roughness and air permeability values of the papers produced from the different cellulosic pulps.

Sample Code	Surface Roughness (ml/min)	Air Permeability (ml/min)
CL	250	2000
CP	220	1800
TD	650	1800
RH	160	1100
CHS	150	900

**Table 15:** Characteristic XRD peak positions observed in the raw, CS and MCS samples.

Sample Stage	Characteristic Peak(s) (2θ, °)	Interpretation
Raw material	16.4/22.6	Cellulose I crystalline structure; broad peaks owing to lignin/hemicellulose
Cellulose sponge (CS)	16.4/22.6 (sharper)	Increased crystalline order owing to delignification
Modified sponge (MCS)	16.4/22.6/~19.2 (new)	Slight structural change owing to surface esterification

**Table 16:** Segal crystallinity index (CrI) calculated from the raw XRD diffractograms at each processing stage.

Sample	Raw CrI (%)	CS CrI (%)	MCS CrI (%)
RH	18.1	42.7	40.1
CHS	–	51.5	71.7
TD	–	65.3	22.6
CL	–	49.8	n.a. ^a^
CP	–	40.0	66.4

^a^ A new, dominant low-angle reflection at 2θ ≈ 12.2° (Figure 19d), attributed to stearate side-chain crystallisation, exceeds the intensity of the residual cellulose 002 peak in MCS-CL, so the conventional Segal formula does not give a physically meaningful crystallinity value for this sample; see text.

**Table 17:** Moisture loss, main-decomposition onset temperature, DTG peak temperature (Tmax) and residue at 800 °C obtained from TGA/DTG analysis of the samples.

Sample	Moisture Loss (%)	Main Decomp. Onset (°C)	DTG Peak Tmax (°C)	Residue at 800 °C (%)
RH (raw)	8.4	220	336	34
CS-RH	6.0	220	336	15
MCS-RH	4.3	216	320	10
CHS (raw)	9.0	200	370	23
CS-CHS	7.0	200	330	17
MCS-CHS	4.0	155	340	9
CS-TD	6.0	230	340	12
MCS-TD	4.8	155	340	13
CS-CP	6.0	250	360	11
MCS-CP	5.0	155	360	14
CS-CL	1.2	250	350	19
MCS-CL	2.3	250	433	15

## Data Availability

The raw data supporting the conclusions of this article will be made available by the authors on request.
